# Selectively nitrogen-doped carbon materials as superior metal-free catalysts for oxygen reduction

**DOI:** 10.1038/s41467-018-05878-y

**Published:** 2018-08-23

**Authors:** Qing Lv, Wenyan Si, Jianjiang He, Lei Sun, Chunfang Zhang, Ning Wang, Ze Yang, Xiaodong Li, Xin Wang, Weiqiao Deng, Yunze Long, Changshui Huang, Yuliang Li

**Affiliations:** 1 Qingdao Institute of Bioenergy and Bioprocess Technology, Chinese Academy of Sciences, No. 189 Songling Road, 266101 Qingdao, China; 2 Qingdao University, 308 Ningxia Road, 266071 Qingdao, China; 3 Dalian Institute of Chemical Physics, Chinese Academy of Sciences, 116023 Dalian, China; 4 Beijing Computational Science Research Center, No.10 East Xibeiwang Road Haidian District, 100193 Beijing, China; 5 Institute of Chemistry, Chinese Academy of Sciences, 100190 Beijing, China; 6 Present Address: Institute of Molecular Sciences and Engineering, Shandong University, No. 72 Binhai Road, 266235 Qingdao, China

## Abstract

Doping with pyridinic nitrogen atoms is known as an effective strategy to improve the activity of carbon-based catalysts for the oxygen reduction reaction. However, pyridinic nitrogen atoms prefer to occupy at the edge or defect sites of carbon materials. Here, a carbon framework named as hydrogen-substituted graphdiyne provides a suitable carbon matrix for pyridinic nitrogen doping. In hydrogen-substituted graphdiyne, three of the carbon atoms in a benzene ring are bonded to hydrogen and serve as active sites, like the edge or defect positions of conventional carbon materials, on which pyridinic nitrogen can be selectively doped. The as-synthesized pyridinic nitrogen-doped hydrogen-substituted graphdiyne shows much better electrocatalytic performance for the oxygen reduction reaction than that of the commercial platinum-based catalyst in alkaline media and comparable activity in acidic media. Density functional theory calculations demonstrate that the pyridinic nitrogen-doped hydrogen-substituted graphdiyne is more effective than pyridinic nitrogen-doped graphene for oxygen reduction.

## Abstract (web summary)

Doping carbon-based materials with nitrogen is effective for enhancing catalytic activity for oxygen reduction; however, directing nitrogen dopants to specific locations is difficult. Here the authors employ hydrogen-substituted graphdiyne as a matrix for nitrogen doping, leading to enhanced performance.

## Introduction

Carbon-based metal-free catalysts are promising alternatives to noble-metals for the oxygen reduction reaction (ORR) due to low cost, abundant reserves and excellent resistance to carbon oxide and methanol^1,2^. Many heteroatom-doped carbon materials demonstrate high activity for ORR. Among those catalysts, nitrogen (N)-doped carbon materials are some of the most efficient metal-free catalysts^3–6^. The radius of the N atom (0.74 Å) is close to that of a carbon (C) atom (0.77 Å), while the electronegativity of N (3.04) is higher than that of C (2.55). Therefore, N atoms are easily doped into carbon materials, and consequently modify the electronic distribution of the carbon-based network. However, the catalytic activities of these carbon-based metal-free catalysts are similar to commercial Pt/C in alkaline medium^3,4,7,8^, and inferior to Pt/C in acidic media^1,9,10^. Thus, it is of importance to prepare carbon-based electrocatalysts with much more efficient active sites for ORR through rational design and skillful preparation.

Generally, three main types of N are doped into carbon materials: pyridinic N, pyrrolic N and graphitic N. Pyridinic N is considered as the most efficient one for ORR among those three^11–15^. Kondo et al. reported recently that the ORR activity was determined by the concentration of pyridinic N^12^. However, for widely researched carbon materials, such as graphene, carbon nanotubes, fullerene and graphite, pyridinic N atoms normally prefer to occupy the edges or defects of the carbon materials^1,12,16,17^. To increase the doping amount of pyridinic N, many methods^18–20^, such as ball-milling, have been implemented to increase the number of edges or defects in carbon materials. However, as the concentration of edges and defects is low in these carbon materials, it is difficult to selectively dope a sufficient amount of pyridinic nitrogen atoms. This is a bottleneck in researching the catalysis mechanism and application of carbon-based materials. Therefore, it is highly desired to explore a new type of carbon framework as the matrix for pyridinic N doping.

Synthesized by Li et al.^21–24^, graphdiyne (GDY) is a special carbon allotrope composed of *sp*^2^ and *sp* hybridized carbon atoms and contains two diacetylenic linkages between benzene rings. The two-dimensional planar structure with a delocalized π-system and large triangular-like pores (diameter ~2.5 Å) of GDY, investigated directly through high-resolution transmission electron microscopy (HRTEM)^25^, are beneficial to the electron transport and mass transfer of reactants and products in ORR. Density functional theory (DFT) calculations indicate that the insertion of the acetylenic linkages creates some positively charged carbon atoms in GDY, which can be active sites for ORR^26–28^. It has been reported that GDY and GDY doped with heteroatoms exhibit high activity for ORR^29–31^.

Inspired by the preferential doping position of pyridinic N in carbon materials and the unique structure of GDY, hydrogen-substituted graphdiyne (HsGDY) was rationally designed and synthesized. The conjugated structure of HsGDY, similar to graphene and GDY, is convenient for electron transfer. Larger molecular pores with a diameter of ca. 16.3 Å are distributed in the HsGDY framework plane, benefiting the mass transfer of O_2_. More importantly, three of the carbon atoms in a benzene ring are linked to acetylenic bonds, while the other three carbon atoms are linked to hydrogen (H) atoms. Accordingly, the C atoms linked to H are easily substituted by N atoms and act similarly to the carbon atoms at the edges or defects of graphene, allowing doping of pyridinic N at “in-plane sites” of the matrix^32,33^ while maintaining the conjugated structure. HsGDY is likely an ideal model carbon framework to selectively dope pyridinic N to obtain highly active metal-free ORR catalysts.

**Fig. 1:**
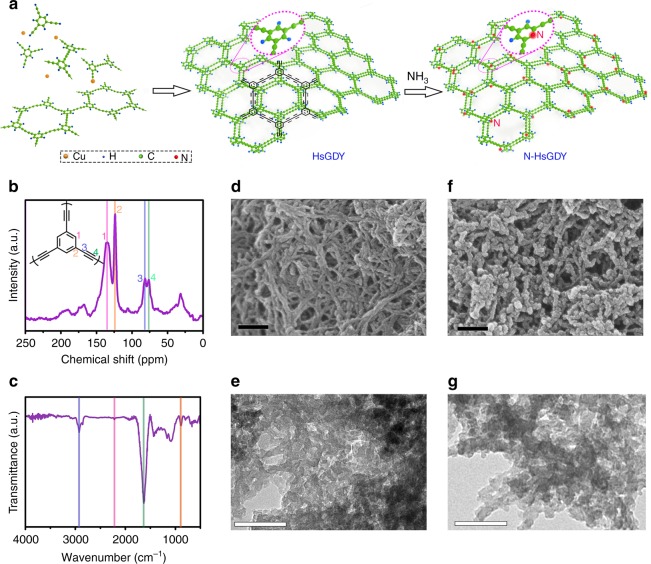
Preparation and characterization of hydrogen-substituted graphdiyne-based materials. **a** Schematic illustration of the preparation process for the hydrogen-substituted graphdiyne (HsGDY) from the monomer triethynylbenzene. Triethynylbenzene molecules are polymerized to generate HsGDY through a cross-coupling reaction with copper ion as catalyst. The HsGDY is pyrolyzed in an ammonia (NH_3_) atmosphere to produce pyridinic nitrogen-doped hydrogen-substituted graphdiyne (N-HsGDY). For clarity, HsGDY and N-HsGDY are shown with only two layers. **b** ^13^C solid-state NMR spectrum for HsGDY; the insert displays the structure of the monomer of HsGDY. **c** Fourier transform infrared spectrum of the HsGDY, **d** scanning electron microscopy (SEM) and **e** transmission electron microscopy (TEM) images of HsGDY. **f** SEM and **g** TEM images of N-HsGDY-900 °C. Scale bar, 100 nm for **d**–**g**

Herein, through a cross-coupling reaction, HsGDY is successfully synthesized on the surface of copper foil from the monomer of triethynylbenzene. The HsGDY is then facilely treated in an ammonia (NH_3_) atmosphere at high temperature to obtain N-doped HsGDY (N-HsGDY) (Fig. 1a), which exhibits outstanding catalytic activity for ORR according to both experiments and theoretical calculations. In N-HsGDY, pyridinic N atoms are selectively doped into HsGDY, as evidenced by X-ray photoelectron spectroscopy (XPS), X-ray absorption near-edge structure (XANES) spectroscopic measurements and DFT calculations. The as-synthesized pyridinic N-HsGDY is demonstrated as one of the most active metal-free catalysts, with activity that is higher than that of a commercially available Pt/C (20 wt%, JM) in alkaline media. The N-HsGDY also exhibits stability and methanol tolerance that is superior to that of Pt/C (JM). Moreover, the catalytic performance of N-HsGDY in acidic media is also demonstrated comparable to that of Pt/C (JM).

## Results

### Synthesis and characterization

The details for the synthesis process of HsGDY and N-HsGDY are described in the Methods section (Supplementary Fig. 1a and Fig. 1a). Briefly, triethynylbenzene was used as the monomer, while copper foil was applied as catalyst and supporter. Large-area HsGDY film was obtained on the surface of copper foil through a cross-coupling reaction. HsGDY film was peeled off from copper foil with hydrochloric acid and rinsed thoroughly to get HsGDY powder. The structure of the monomer, triethynylbenzene, was characterized by ^1^H nuclear magnetic resonance (NMR) (Supplementary Fig. 2) and ^13^C NMR spectra (Supplementary Fig. 3). Fourier transform infrared spectroscopy (FT-IR) data (Supplementary Fig. 4) and Raman spectra (Supplementary Fig. 5) for triethynylbenzene also confirmed the presence of aromatic ring units and acetylenic bonds in HsGDY.

The structure of HsGDY was verified by ^13^C solid-state NMR, XPS, and FT-IR. In the NMR spectrum, the peaks at 123.7 ppm and 135.2 ppm correspond to aromatic C-C and C-H sites, while the peaks at 76.0 ppm and 81.7 ppm belong to C(*sp*)-C(*sp*) and C(*sp*)-C(*sp*^2^) sites (Fig. 1b). The ratio of C(*sp*)-C(*sp*) and C(*sp*)-C(*sp*^2^) is close to 1: 1, as calculated from the deconvoluted areas of the C 1*s* region in the XPS spectra (Supplementary Fig. 6). FT-IR spectra also demonstrated the aromatic C-C and C-H structure existing in HsGDY and a small peak at ca. 2210 cm^−1^ corresponds to C≡C (Fig. 1c). The morphology of HsGDY was observed using scanning electron microscopy (SEM) (Fig. 1d) and transmission electron microscopy (TEM) (Fig. 1e) images. It can be seen that the HsGDY nanowires are intertwined together to form a three-dimensional (3D) porous structure.

The as-synthesized HsGDY was then treated in NH_3_ atmosphere at 700 °C, 800 °C, 900 °C and 1000 °C to gain N-HsGDY-700 °C, N-HsGDY-800 °C, N-HsGDY-900 °C and N-HsGDY-1000 °C, respectively (Fig. 1a). The SEM images of all the N-HsGDY catalysts are shown in Fig. 1f and Supplementary Fig. 7. It can be found that after the doping of N at high temperatures, N-HsGDY materials almost maintain the morphology of HsGDY, except that some nanowires are agglomerated when the treatment temperature higher than 900 °C. The 3D porous structure of these catalysts is advantageous for the mass transfer of oxygen and water in ORR. The porous structure of all the catalysts can also be observed from the TEM images (Fig. 1g and Supplementary Fig. 8), consistent with the SEM results. In the ^13^C solid-state NMR spectrum of N-HsGDY-900 °C, the peaks for carbon atoms in aromatic rings and butadiyne can be observed like the spectrum of HsGDY, except that a new small peak for aromatic ring carbon at ~132 ppm appears because of the doping of pyridinic N in some aromatic rings (Supplementary Fig. 9b). The degree of ordering of the catalysts were determined by the Raman spectrum (Supplementary Fig. 10). D and G peaks located at ca. 1333 and 1587 cm^−1^ are assigned to “defects” and ordered carbon, respectively. The intensity ratio of *I*_D_/*I*_G_ increases largely for N-HsGDY-700 °C, compared to HsGDY, indicating that the substitution of N atoms induces many structural defects in HsGDY framework. The ratios of *I*_D_/*I*_G_ decrease gradually with the increase of calcined temperatures from 700 °C to 1000 °C, indicating that the structure of N-HsGDY samples becomes ordered after treatment at high temperature under NH_3_ atmosphere (Supplementary Figs 10 and 11), which can improve the electrical conductivity. Besides, there is a small peak at ca. 2156 cm^−1^ in the Raman spectrum that corresponds to C≡C, demonstrating the existence of C≡C in HsGDY and each N-HsGDY sample. FT-IR spectra also support that the N-HsGDY maintains the major carbon structure of HsGDY (Supplementary Fig. 12).

**Fig. 2:**
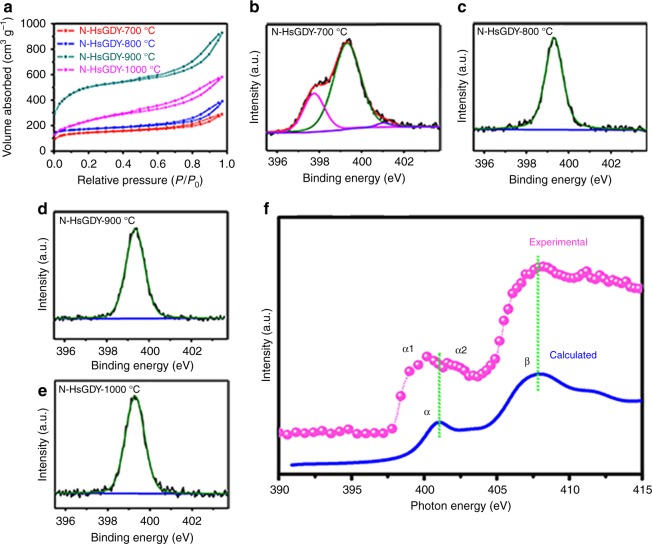
Structural information for pyridinic nitrogen-doped hydrogen-substituted graphdiyne. **a** Nitrogen adsorption/desorption isotherms for all of the pyridinic nitrogen-doped hydrogen-substituted graphdiyne (N-HsGDY) catalysts. **b**–**e** High-resolution X-ray photoelectron spectroscopy (XPS) spectra of N 1*s* for N-HsGDY treated at 700 °C (N-HsGDY-700 °C) (**b**), N-HsGDY treated at 800 °C (N-HsGDY-800 °C) (**c**), N-HsGDY treated at 900 °C (N-HsGDY-900 °C) (**d**), and N-HsGDY treated at 1000 °C (N-HsGDY-1000 °C) (**e**). **f** The experimental and calculated curves of N K-edge X-ray absorption near-edge structure (XANES) spectra for N-HsGDY-900 °C

Further, N_2_ adsorption–desorption analysis was used to determine the surface area and pore size distribution of the catalysts (Fig. 2a and Supplementary Fig. 14). It is calculated that the Brunauer–Emmett–Teller (BET) surface areas of HsGDY, N-HsGDY-700 °C, N-HsGDY-800 °C, N-HsGDY-900 °C and N-HsGDY-1000 °C are 486 m^2^ g^−1^, 535 m^2^ g^−1^, 650 m^2^ g^−1^, 1754 m^2^ g^−1^ and 780 m^2^ g^−1^, respectively. The surface areas become larger with an increase in heat-treatment temperature and reach the highest value when the temperature is around 900 °C. The smaller surface area of N-HsGDY-1000 °C should be derived from the agglomeration of HsGDY nanowires, as shown in the SEM (Supplementary Fig. 7e) and TEM images (Supplementary Fig. 8e). The pore-size distribution curves are shown in Supplementary Fig. 14. Many micropores around 1.6 nm can be observed, which is consistent with the porous plane structure and pore diameter of HsGDY (Supplementary Fig. 1).

XPS spectra reveal that N atoms are successfully introduced into the structure of HsGDY for N-HsGDY catalysts. The contents of N are 3.5 ± 0.3 at%, 2.8 ± 0.3 at%, 2.9 ± 0.3 at% and 2.8 ± 0.3 at% for N-HsGDY-700 °C, N-HsGDY-800 °C, N-HsGDY-900 °C, and N-HsGDY-1000 °C, respectively, demonstrated by the XPS survey spectrum (Supplementary Fig. 15). It is interesting to find that only one symmetrical peak exists at ca. 399.3 eV in the high-resolution N1*s* spectrum of N-HsGDY, excluding N-HsGDY-700 °C (Fig. 2b-e), which should be attributed to pyridinic N^34,35^. The configuration of the doped N in N-HsGDY-900 °C was further confirmed by XANES (Fig. 2f). The N K-edge of pyridinic N-HsGDY (Supplementary Fig. 16) was also simulated by DFT methods. As shown by the blue line in Fig. 2f, peak α is the N 1*s*-π* transition of the pyridinic N^36^, while peak β is the N 1s-σ* transition. The experimental result is strongly consistent with the simulation curve, except that the α peak is split into double peaks, demonstrating only pyridinic N existed in N-HsGDY-900 °C. The double peaks (α_1_ and α_2_) may be caused by the different positions of pyridinic N atoms, at the edge or inside of HsGDY^37^.

**Fig. 3:**
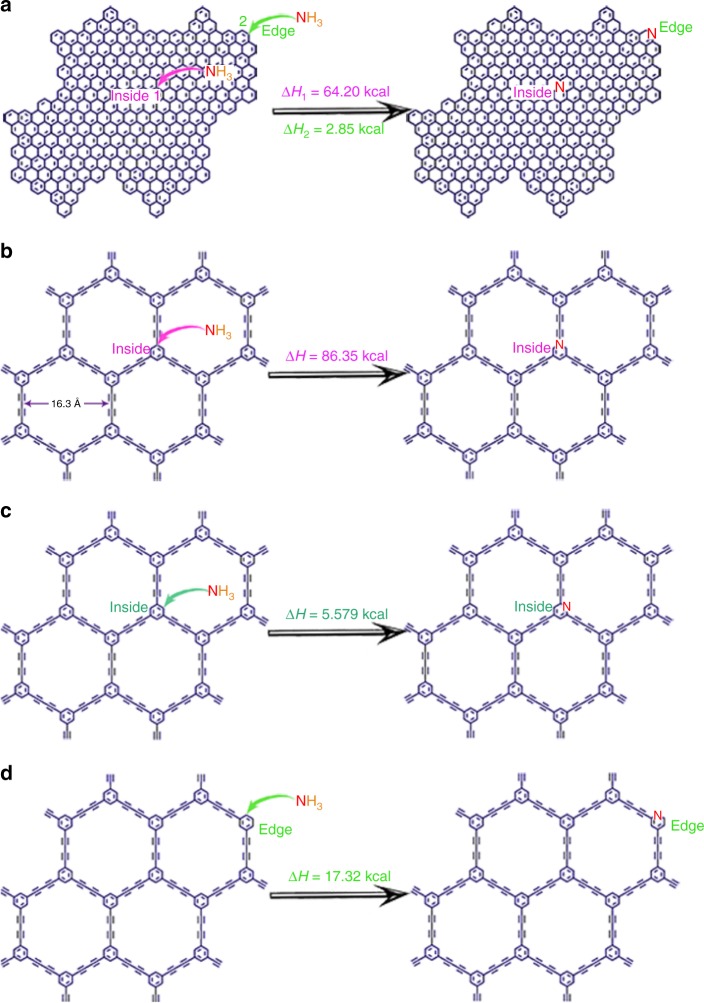
Reaction equations and enthalpy changes. **a** A reaction equation and enthalpy changes for substituting C in graphene with N. **b** A reaction equation and enthalpy change for substituting C linked to a acetylenic bond in hydrogen-substituted graphdiyne (HsGDY) with graphitic N. **c** A reaction equation and enthalpy change for substituting C on the inside of HsGDY with pyridinic N. **d** A reaction equation and enthalpy change for substituting C at the edge of HsGDY with pyridinic N. The C in the benzene ring at the edge of HsGDY was linked with H (**d**)

This selective doping of pyridinic N in N-HsGDY can be explained by the calculated enthalpy changes of N substituting C in HsGDY. For the traditional carbon materials, such as graphene, pyridinic N is preferably generated at the edge. As shown in Fig. 3a, less energy is needed to substitute C at the edge (Δ*H* = 2.85 kcal) than that on the inside (Δ*H* = 64.20 kcal) to form pyridinic N in graphene. However, as for HsGDY, the enthalpy changes for these N doping reactions are different. As shown in Fig. 3b, c, much more energy is needed to substitute carbon atoms linked to acetylenic carbon and form graphitic N (Δ*H* = 86.35 kcal) than that to substitute carbon atoms and form pyridinic N (Δ*H* = 5.579 kcal) on the inside of HsGDY. Two possible edge structures of the HsGDY, the benzene ring at the edge of HsGDY linked with hydrogen or alkynyl, were considered in the calculations (Fig. 3d and Supplementary Fig.17). Both results indicate that the calculated enthalpy changes for N substituting C linked to H on the inside and at the edge of HsGDY are close (Fig. 3c, d and Supplementary Fig.17). Therefore, pyridinic N is easy to form, not only at the edge but also on the inside of HsGDY, showing that HsGDY provides abundant active positions similar to the edge or defect sites. Thus, rational design of HsGDY as a matrix allows for selective pyridinic N doping. As pyridinic N is the most effective doping configuration to improve the catalytic activity for ORR^12^, these selectively doped pyridinic N sites in HsGDY should deliver high catalytic activity for ORR. Although some imine N (397.7 eV) and pyrrolic/quaternary N (401 eV) exist in N-HsGDY-700 °C^29,30^, only pyridinic N sites are selectively added with processing temperatures above 800 °C (Fig. 2b–f), possibly due to a higher stability of pyridinic N than imine N and pyrrolic/quaternary N at high temperature. The C 1*s* region of XPS spectra (Supplementary Fig. 18) can be deconvoluted into four Gaussian fractions at ∼284.6, 285.2, 286.6 and 288.8 eV, corresponding to C–C (*sp*^2^), C-C (*sp*), C-O and C=O, respectively, for HsGDY^24,38,39^. An extra peak at 286.2 eV is attributed to C=N for the four N-HsGDY catalysts, demonstrating that N atoms were doped into the HsGDY materials. The content ratios of *sp*^2^ C: *sp* C on the surface of the catalysts were determined by the area ratios of these two bands which are close to 1:1 for all the four N-HsGDY, consistent with the structure of HsGDY. This indicates that the structure is maintained after doping N at high temperature for N-HsGDY catalysts.

### Electrochemical evaluation in alkaline media

**Fig. 4:**
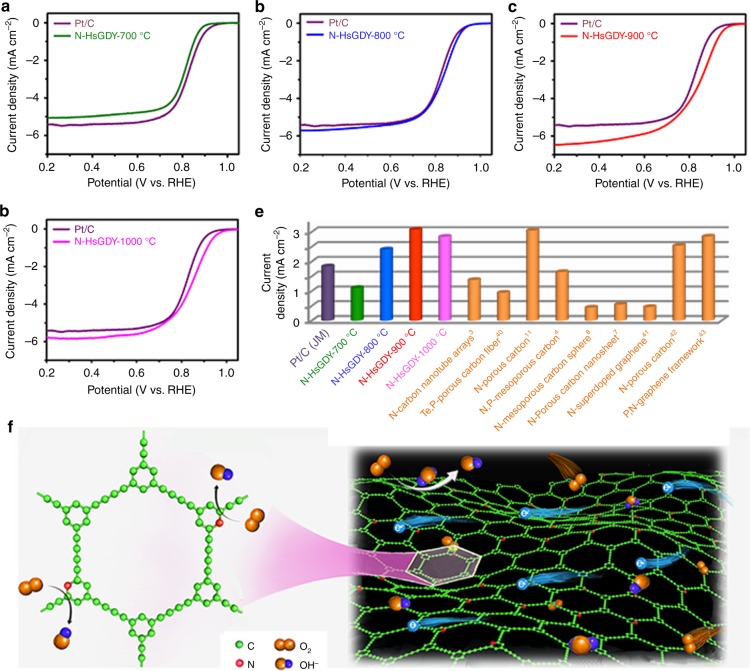
Performance of catalysts in alkaline media. **a**–**d** Linear sweep voltammetry curves of pyridinic nitrogen-doped hydrogen-substituted graphdiyne (N-HsGDY) treated at 700 °C (N-HsGDY-700 °C) (**a**), N-HsGDY treated at 800 °C (N-HsGDY-800 °C) (**b**), N-HsGDY treated at 900 °C (N-HsGDY-900 °C) (**c**), and N-HsGDY treated at 1000 °C (N-HsGDY-1000 °C) (**d**), compared to commercial carbon-based platinum catalyst (Pt/C (JM)) for the oxygen reduction reaction (ORR). **e** Comparison of current density at 0.85 V vs. reversible hydrogen electrode (RHE) of Pt/C, N-HsGDY catalysts in this work and previously reported metal-free catalysts^3,4,7,8,11,40–43^. **f** Schematic diagram for the catalytic process for ORR with N-HsGDY

To evaluate the ORR performance of the N-HsGDY catalysts in alkaline medium, rotating disk electrode (RDE) measurements were conducted. First, the as-synthesized undoped HsGDY was used as catalyst for ORR. The LSV curves indicate that the HsGDY can catalyze the ORR, although its catalytic activity is much lower than that of commercial Pt/C (JM) (Supplementary Fig. 19). The catalytic activity of HsGDY may be derived from the positively charged carbon atoms because of the insertion of acetylenic linkage in HsGDY, similar to GDY^27^. The catalytic activities of N-doped HsGDYs are improved greatly (Fig. 4a–d). The activity of N-HsGDY-800 °C, N-HsGDY-900 °C, and N-HsGDY-1000 °C are all higher than Pt/C (JM) at all measured potentials between 0.2 and 1.1 V vs. RHE (Fig. 4b–d). In those N-HsGDYs, all N atoms are doped as pyridinic N (Fig. 2b–f). As described above, pyridinic N is highly efficient to create active sites for ORR, thus the high activities of N-HsGDY-800 °C, N-HsGDY-900 °C, and N-HsGDY-1000 °C should be related to the doped pyridinic N. The current densities at 0.85 V vs. RHE of Pt/C (1.79 mA cm^−2^), N-HsGDY-700 °C (1.06 mA cm^−2^), N-HsGDY-800 °C (2.34 mA cm^−2^), N-HsGDY-900 °C (3.03 mA cm^−2^), and N-HsGDY-1000 °C (2.79 mA cm^−2^) are compared with some recently reported carbon-based metal-free catalysts, as shown in Fig. 4e. Among them, N-HsGDY-900 °C displays the best catalytic activity, which should be caused by the high concentration of pyridinic N and large BET surface areas, as shown in Fig. 2a. For N-HsGDY-900 °C, the onset potential reaches ca. 1.02 V vs. RHE, while the half-wave potential (E_1/2_) is ca. 0.85 V vs. RHE and limited current density is ca. 6.2 mA cm^−2^, which are among the best reported to date^3,4,11,29,38,40–44^ (Fig. 4e and Supplementary Table 2). The current density of N-HsGDY-900 °C at 0.85 V vs. RHE is 1.6 times higher than that of commercial Pt/C (JM). According to previously reported literature, pyridinic N can create positively charged carbon atoms, which are active sites for ORR^12,45,46^. Besides, the conjugated structures of HsGDY and N-HsGDY benefit electron transfer, while the pore structure contributes to mass transfer of O_2_ and H_2_O.

**Fig. 5:**
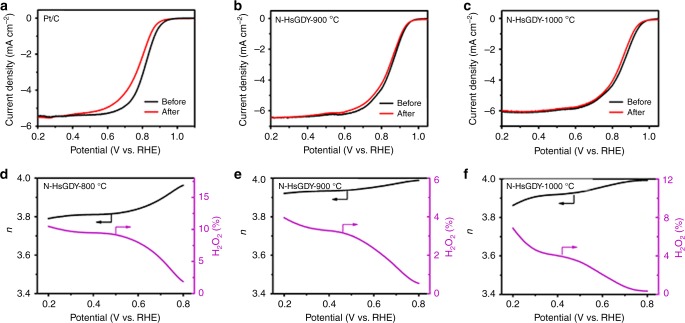
Stability and electron transfer number of catalysts for oxygen reduction in basic media. **a**–**c** Durability tests of the commercial carbon-based platinum catalyst (Pt/C) (**a**), pyridinic nitrogen-doped hydrogen-substituted graphdiyne (N-HsGDY) treated at 900 °C (N-HsGDY-900 °C) (**b**), and N-HsGDY treated at 1000 °C (N-HsGDY-1000 °C) (**c**) catalysts before and after 5000 cycles in O_2_-saturated 0.1 M KOH. **d**–**f** Electron transfer numbers and peroxide yields calculated from the rotating ring-disk electrode (RRDE) measurements for N-HsGDY treated at 800 °C (N-HsGDY-800 °C) (**d**), N-HsGDY-900 °C (**e**), and N-HsGDY-1000 °C (**f**)

Moreover, the stabilities of the N-HsGDY catalysts are excellent as well, as demonstrated by the potential shifts after rapid aging tests in O_2_-saturated 0.1 M KOH solution. A negative shift of 35 mV in E_1/2_ can be observed for the Pt/C catalyst (Fig. 5a). However, the LSV curves show negligible variation for N-HsGDY-800 °C (Supplementary Fig. 23), N-HsGDY-900 °C (Fig. 5b) and N-HsGDY-1000 °C (Fig. 5c). The stabilities of these catalysts were also measured by chronoamperometric (CA) measurements at 0.7 V vs. RHE in O_2_-saturated 0.1 M KOH solution. The current decays after 10 hours of N-HsGDY-800 °C, N-HsGDY-900 °C and N-HsGDY-1000 °C are all obviously lower than that of Pt/C (JM) (Supplementary Fig. 24), indicating N-HsGDY catalysts are more stable than Pt/C (JM). Methanol tolerances of the catalysts were tested by CA measurements, in which methanol was injected into the KOH solution at 400 s (Supplementary Fig. 25). The activity decays of N-HsGDY-800 °C, N-HsGDY-900 °C and N-HsGDY-1000 °C are all much lower than that of Pt (JM). These results indicate that N-HsGDY-800 °C, N-HsGDY-900 °C, and N-HsGDY-1000 °C have much better stability and methanol tolerance than that of Pt/C (JM). The reaction pathways can be estimated from the electron transfer number (*n*) derived from the RRDE tests (see the Methods section for details). The electrons transfer numbers were calculated to be 3.8–4.0 for these three N-HsGDY catalysts from RRDE measurements, suggesting that ORRs were mainly conducted by four-electron transfer pathway on these catalysts. A H_2_O_2_ yield of less than 10% was observed during the reaction process (Fig. 5d–f). It should be noted that N-HsGDY-900 °C catalyst exhibits the highest activity, stability and methanol tolerance; and the electron transfer number is above 3.92 with a H_2_O_2_ yield lower than 4%. N-HsGDY-900 °C is a superior electrocatalyst for ORR. Furthermore, the Tafel slope of N-HsGDY-900 °C, recorded from the LSV tests (Fig. 4c), is smaller than that of Pt/C (JM) (64.4 mV dec^-1^ for N-HsGDY-900 °C and 72.6 mV dec^-1^ for Pt/C, Supplementary Fig. 26), further demonstrating the excellent electrocatalytic activity of N-HsGDY-900 °C. Electrochemical impedance spectroscopy (EIS) was used to study the charge transfer resistance of the catalysts (Supplementary Fig. 27). The results reveal that N-HsGDY-900 °C has the smallest charge transfer resistance among the four N-HsGDY catalysts.

### Electrochemical evaluation in acidic media

**Fig. 6:**
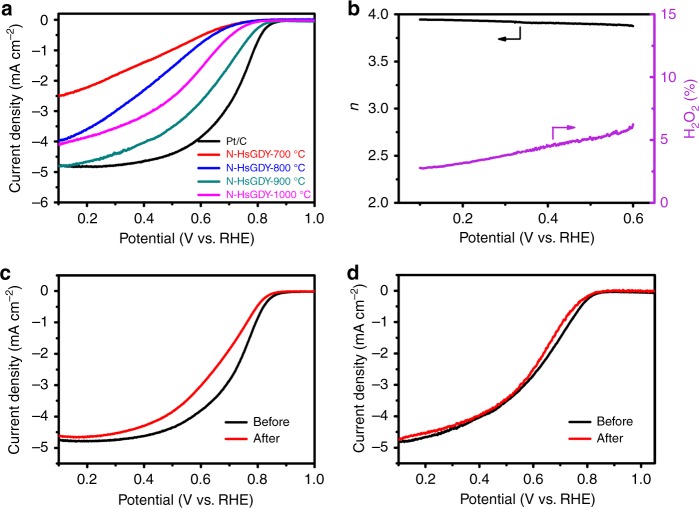
Performance of catalysts for the oxygen reduction reaction in acidic media. **a** Linear sweep voltammetry curves of commercial Pt/C and all of the pyridinic nitrogen-doped hydrogen-substituted graphdiyne (N-HsGDY) catalysts recorded in O_2_-saturated 0.1 M HClO_4_ at 1600 rpm. **b** Electron transfer number and peroxide yield calculated from the rotating ring-disk electrode (RRDE) measurements for N-HsGDY treated at 900 °C (N-HsGDY-900 °C). **c**, **d** Durability tests of the commercial carbon-based platinum catalyst (Pt/C) (**c**), and N-HsGDY-900 °C (**d**) catalysts before and after 5000 cycles in O_2_-saturated 0.1 M HClO_4_

The performance of the N-HsGDY catalysts in acidic media were also measured in 0.1 M HClO_4_ solution. The LSV tests indicate that the catalytic activity of N-HsGDY-900 °C is comparable to that of Pt/C (JM) with an onset potential at 0.86 V vs. RHE and a half-wave potential at 0.64 V vs. RHE, higher than that of other N-HsGDY catalysts (Fig. 6a) due to the concentration of pyridinic N and surface area. The catalytic activity is high in comparison with recently reported metal-free catalysts for ORR in acidic media (Supplementary Table 3). The electron transfer number of N-HsGDY-900 °C is among 3.88–3.95 at potentials between 0.1 and 0.6 V vs. RHE (Fig. 6b). The yield of peroxide is below 6%, showing that ORR mainly occurs by a four-electron transfer pathway on N-HsGDY-900 °C in acidic media. The stability of the catalysts was tested by measuring the LSVs before and after cyclic voltammetry (CV) in O_2_-saturated HClO_4_ for 5000 cycles (Fig. 6c, d). Although the LSV curves of Pt/C (JM) changed significantly, the change for N-HsGDY-900 °C is negligible after a CV test for 5000 cycles, which indicates that N-HsGDY-900 °C is more stable than Pt/C (JM). The methanol tolerance of the catalysts in acidic media was also measured by a CA test with methanol injected at 400 s. The current decays ca. 13% for Pt/C (JM) after the injection of methanol, while the activity of N-HsGDY-900 °C is not affected by the methanol (Supplementary Fig. 28). N-HsGDY-900 °C has better methanol tolerance than Pt/C (JM). The Tafel slope for for N-HsGDY-900 °C in acidic media is 76.7 mV dec^−1^, comparable to that of Pt/C (64.2 mV dec^−1^) (Supplementary Fig. 29).

### Density functional theory calculations

**Fig. 7:**
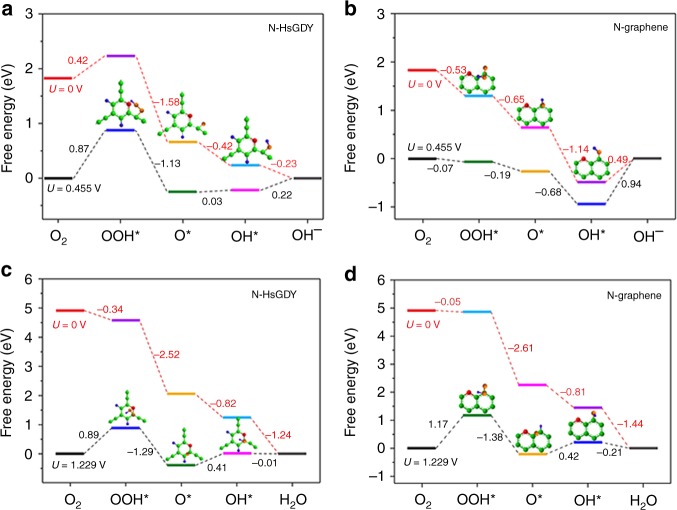
Mechanistic study for the oxygen reduction reaction. **a**, **b** Free energy diagrams of the oxygen reduction reaction (ORR) on pyridinic nitrogen-doped hydrogen-substituted graphdiyne (N-HsGDY) (**a**) and pyridinic N-doped graphene edge (N-graphene) (**b**) at 0.455 and 0 V (vs. normal hydrogen electrode, NHE) in alkaline media. **c**, **d** Free energy diagrams of ORR on pyridinic N-doped HsGDY (**c**) and pyridinic N-doped graphene edge (**d**) at 1.229 and 0 V (vs. NHE) in acidic media

To better understand the extraordinary catalytic activity of pyridinic N-doped HsGDY, the free energy diagrams of pyridinic N-HsGDY and pyridinic N-graphene for ORR in alkaline media at zero electrode potential (*U* = 0 V vs. NHE) and equilibrium potential (*U* = 0.455 V vs. NHE) were calculated using DFT and are shown in Fig. 7a, b and Supplementary Fig. 30. The ORR is composed of four elementary steps:

1 $${\mathrm{O}}_2\left( g \right) + {\mathrm{H}}_2{\mathrm{O}}\left( l \right) + {\mathrm{e}}^ - + ^{\ast} \to {\mathrm{OOH}} ^{\ast} + {\mathrm{OH}}^ - $$

2 $${\mathrm{OOH}} ^{\ast} + {\mathrm{e}}^ - \to {\mathrm{O}} ^{\ast} + {\mathrm{OH}}^ - $$

3 $${\mathrm{O} ^{\ast} + H_2O}\left( l \right) + {\mathrm{e}}^ - \to {\mathrm{OH}} ^{\ast} + {\mathrm{OH}}^ - $$

4 $${\mathrm{OH}} ^{\ast} + {\mathrm{e}}^ - \to ^{\ast} + {\mathrm{OH}}^ - $$

The corresponding atomic configurations of ORR intermediate states are shown in Supplementary Fig. 31. It can be seen that O_2_ molecules cannot be directly adsorbed on both pyridinic N-HsGDY and pyridinic N-graphene, consistent with previous reports^47,48^. For pyridinic N-HsGDY, the acetylenic carbon atom that adjacent to benzene ring is the active site of OOH*, O*, and OH*. The alkynyl groups, inserted between benzene rings for N-HsGDY, induce a relatively higher positive charge density (0.694) for the adjacent C atoms of N than those in N-graphene (0.392) (Supplementary Fig. 32). Although the C atoms adjacent to N are not adsorption active sites due to steric hindrance, the reaction active C atoms are influenced by these positively charged adjacent C. The diverse charge distributions of N-HsGDY and N-graphene lead to different ORR rate-determining steps for the two catalysts. For pyridinic N-HsGDY, the adsoption of OOH* is the most sluggish step with the highest uphill free energy change. As for pyridinic N-graphene, the rate-determining step is the last one, desorption of OH*. Comparing the free energy changes of the rate-determining step for these two catalysts, it is obvious that the resistance to ORR on pyridinic N-HsGDY is smaller than that of pyridinic N-graphene at both 0.455 V (0.87 vs. 0.94 eV) and 0 V (0.42 vs. 0.49 eV) in alkaline media.

In acidic media, the elementary steps are:

5 $${\mathrm{O}}_2\left( g \right) + {\mathrm{H}}^ + + {\mathrm{e}}^ - + ^{\ast} \to {\mathrm{OOH}} ^{\ast} $$

6 $${\mathrm{OOH}} ^{\ast} + {\mathrm{H}}^ + + {\mathrm{e}}^ - \to {\mathrm{O}} ^{\ast} + {\mathrm{H}}_2{\mathrm{O}}\left( l \right)$$

7 $${\mathrm{O}} ^{\ast} + {\mathrm{H}}^ + + {\mathrm{e}}^ - \to {\mathrm{OH}} ^{\ast}$$

8 $${\mathrm{OH}} ^{\ast} + {\mathrm{H}}^ + + {\mathrm{e}}^ - \to {\mathrm{H}}_2{\mathrm{O}}\left( l \right) + ^{\ast} $$

The pyridinic N can adsorb H, which influences the atomic configurations of ORR intermediate states (Supplementary Fig. 33). At *U* = 0 V vs. NHE, all the electron-transfer steps are exothermic for both pyridinic N-HsGDY and pyridinic N-graphene, and thus the free energy pathways are downhill. However, at *U* = 1.229 V vs. NHE, the first step($O_2\left( g \right) {\mathrm{ +} H}^ + + e^ - + ^{\ast} \to {\mathrm{OOH}} ^{\ast}$) and third step (${\mathrm{O}} ^{\ast} {\mathrm{ + }H}^ + {\mathrm{ + }}e^ - \to {\mathrm{OH}} ^{\ast}$) are endothermic. The first step is the rate-determining step for both catalysts. Similar to that in alkaline media, the free energy change of the rate-determining step for pyridinic N-HsGDY (0.89 eV) is smaller than that for pyridinic N-graphene (1.17 eV) at equilibrium potential (Fig. 7c, d). It indicates that the pyridinic N in HsGDY is more effective for ORR than that in graphene, which can explain the extraordinary catalytic activity of pyridinic N-HsGDY.

## Discussion

We have reported the facile and precise synthesis of pyridinic N selectively doped metal-free catalysts for ORR, using a carbon material HsGDY. Half of the carbon atoms in the benzene ring are linked to hydrogen atoms in HsGDY, which provide abundant preferential substitutional slots for generating pyridinic N. Demonstrated by XPS and XANES measurements, pyridinic N was selectively doped into HsGDY by calcining the as-synthesized HsGDY in NH_3_ atmosphere over 800 °C. These pyridinic N doped HsGDY catalysts exhibit superior activity for ORR with higher onset potential, half-wave potential and limited current density than that of commercial Pt/C (JM). Especially, N-HsGDY-900 °C expresses one of the highest activities among reported metal-free catalysts to date in alkaline media and good activity in acidic media. The extraordinary catalytic activity of N-HsGDY was explained by the DFT calculations, which indicated pyridinic N in HsGDY was more effective than that in graphene for ORR. Furthermore, the stability and methanol tolerance of N-HsGDY are better than commercial Pt/C (JM). The ORR mainly proceeds with a four-electron transfer pathway on these N-HsGDY catalysts. N-HsGDYs are excellent electrocatalyts for ORR. This study will open a different avenue for developing pyridinic N selectively doped carbon materials for fuel cells and other energy storage device.

## Methods

### Synthesis of pyridinic nitrogen-doped hydrogen-substituted graphdiyne

HsGDY was synthesized according to ref. ^49^ as we reported, but with some modifications here. Tribromobenzene (4 mmol), ethynyltrimethylsilane ((CH_3_)_3_SiC≡CH, 40 mmol), bis(triphenylphosphine)palladium(II) dichloride (PdCl_2_(PPh_3_)_2_, 0.400 mmol), copper(I) iodide (CuI, 0.8 mmol) and triphenylphosphine (Ph_3_P) (0.8 mmol) were added into triethylamine, successively, and stirred at 80 °C for 5 days. Then the solvent was evaporated and the residue was purified by column chromatography to yield white powder of Tris[(trimethylsilyl)ethynyl]benzene. After that 0.4 mmol of tetrabutylammonium fluoride was mixed with 0.133 mmol tris[(trimethylsilyl)ethynyl]benzene in tetrahydrofuran solution. The mixture was stirred for 30 min at 60 °C and then diluted with CH_2_Cl_2_ and washed with H_2_O. The solvent was removed by vacuum rotary evaporation to yield monomer of HsGDY.

This monomer of HsGDY was dissolve in pyridine and added slowly to another pyridine solution with copper foils at 60 ^o^C. Then the mixture was stirred at 60 ^o^C for 3 days. The obtained copper foil was immersed in concentrated HCl aqueous solution to strip HsGDY film from copper foil. Then the HsGDY was washed with dimethylformamide (DMF) and acetone for three times, and immersed in 3 M HCl and 3 M NaOH for 3 h at 80 °C, successively. After that, the product was washed with abundant H_2_O to get HsGDY powder.

The as-synthesized HsGDY was calcined at 400 °C in Ar atmosphere, and then in NH_3_ atmosphere at 700 °C, 800 °C, 900 °C and 1000 °C, respectively, for 1 h to dope N into HsGDY, gaining N-HsGDY-700 °C, N-HsGDY-800 °C, N-HsGDY-900 °C and N-HsGDY-1000 °C, respectively.

### Electrochemical measurements

A standard three-electrode cell was used for the electrochemical tests. A saturated calomel electrode (SCE) was used as a reference electrode, a Pt plate was used as a counter electrode and the catalyst film coated rotating ring-disk electrode (RRDE, 4 mm in diameter) was used as the working electrode. To prepare the catalyst ink, 5 mg of catalyst was ultrasonically dispersed in a solution of 50 μL Nafion (5 wt %) and 950 μL ethanol. The catalyst ink was cast onto a glassy carbon electrode. The catalyst loadings on RRDE were 0.4 mg cm^−2^ for metal-free catalysts and 20 μg_Pt_ cm^−2^ for commercial Pt/C catalyst. 0.1 M KOH solution and 0.1 M HClO_4_ were used as alkaline and acidic media, respectively, for ORR measurement. The potentials are presented with respect to the reversible hydrogen electrode (RHE). The conversion factors for SCE to RHE are 0.998 V in 0.1 M KOH solution and 0.304 V in 0.1 M HClO_4_, and were acquired by measuring the voltage between the SCE and a Pt-black-coated Pt wire that was immersed in the same electrolyte saturated with H_2_. To saturate the electrolyte with Ar or O_2_, the electrolyte was bubbled with Ar or O_2_ for 20 min prior to each experiment. To ensure O_2_ saturation during the linear sweep voltammetry measurement, O_2_ was flowed through the electrolyte. Cyclic voltammetry experiments were conducted with a scan rate of 50 mV s^−1^ between 0 and 1.2 V vs. RHE at room temperature. The catalytic activities were determined using linear sweep voltammetry (LSV) from 0 to 1.2 V at a scan rate of 5 mV s^−1^ at different rotation rates, while the ring electrode was held at 1.3 V vs. RHE. The current densities were normalized to the geometry area of the glassy carbon disk in the LSV curves in this paper. The H_2_O_2_ collection coefficient at the ring in RRDE experiments was 0.37, as measured using a Fe(CN)_6_^4-/3-^ redox couple. All the ORR currents presented in the figures are Faradaic currents, i.e. after correction for the capacitive current. The RRDE measurements were also carried out to determine the electron transfer number of ORR. The following equations were used to calculate *n* (the apparent number of electrons transferred during ORR) and %H_2_O_2_ (the percentage of H_2_O_2_ released during ORR),

9 $${\mathrm{n}} = \frac{{4I_{\mathrm D}}}{{I_{\mathrm D} + \left( {I_{\mathrm R}/N} \right)}}$$

10 $${\% }{\mathrm {H}}_2{\mathrm {O}}_2 = 100\frac{{2I_{\mathrm R}/N}}{{I_{\mathrm D} + \left( {I_{\mathrm R}/N} \right)}}$$

where *I*_D_ is the Faradaic current at the disk, *I*_R_ the Faradaic current at the ring and *N* is the H_2_O_2_ collection coefficient at the ring. The apparent number of electrons transferred for ORR on the electrodes was also determined by the Koutechy–Levich equation given blow:

11 $$\frac{1}{j} = \frac{1}{{j_{\mathrm L}}} + \frac{1}{{j_{\mathrm k}}} = \frac{1}{{B\omega ^{1/2}}} + \frac{1}{{j_{\mathrm k}}}$$

12 $${B} = 0.62{nF}C_0\left( {D_0} \right)^{2/3}\nu ^{ - 1/6}$$

where *j* is measured current density, *j*_k_ is kinetic current density, *j*_L_ is diffusion limited current density, *ω* is electrode rotation rate, *F* is Faraday constant (96,485 C mol^−1^), *C*_0_ is bulk concentration of O_2_ (1.2 × 10^−3^ mol L^−1^ for both 0.1 M KOH solution and 0.1 M HClO_4_ solution), *D*_0_ is diffusion coefficient of O_2_ (1.9 × 10^−5^ cm^2^ s^−1^ for 0.1 M KOH solution and 1.93 × 10^−5^ cm^2^ s^−1^ for 0.1 M HClO_4_ solution) and *ν* is kinetic viscosity of the electrolyte (0.01 cm^2^ s^−1^ for both 0.1 M KOH solution and 0.1 M HClO_4_ solution)^50^.

Tafel slope was calculated according to Tafel equation:

13 $$E = a + b \,{\mathrm log}\,\left( {j_{\mathrm k}} \right)$$

Where *E* is the applied potential in the LSV test, *a* is a constant, *b* is the Tafel slop and *j*_k_ is the kinetic current density.

The stability tests were conducted by continuous cyclic voltammetry between 0.6 and 1.2 V (vs. RHE) in O_2_-saturated 0.1 M KOH or HClO_4_ solution with scan rate of 100 mV s^−1^ for 5000 cycles. The stability was determined by measuring the change of LSVs before and after the cyclic voltammetry. Chronoamperometric (CA) measurements were also used to judge the stability of the catalysts with the potential holding at 0.7 V (vs. RHE) in O_2_-saturated 0.1 M KOH electrolyte and 0.65 V (vs. RHE) in O_2_-saturated 0.1 M HClO_4_ electrolyte at a rotating rate of 400 rpm.

Methanol tolerance experiments were developed with CA measurements at 0.8 V (vs. RHE)/0.65 V (vs. RHE) in O_2_-saturated 0.1 M solution at rotating rate of 1600 rpm. A volume of 4 mL methanol was injected into the KOH/HClO_4_ solution at ca. 400 s.

Electrochemical impedance spectroscopy (EIS) was used to study the charge transfer resistance of the catalysts. EIS tests were conducted in O_2_-saturated 0.1 M KOH with the rotating rate of 1600 rpm.

All the electrochemical tests were carried out at ambient temperature.

### Data availability

The data that support the findings of this study are available from the corresponding author on request.

## Electronic supplementary material

Supplementary Information
