# Supplementary material for: Selectively nitrogen-doped carbon materials as superior metal-free catalysts for oxygen reduction
Source: Nat Commun. 2018 Aug 23;9:3376. doi: 10.1038/s41467-018-05878-y (PMC6107639; doi:10.1038/s41467-018-05878-y)
Supplement: Supplementary file 1 — Supplementary Information [file 41467_2018_5878_MOESM1_ESM.pdf]

Supplementary Information for

Selectively nitrogen doped carbon materials as superior metal-free catalysts for oxygen reduction

Lv *et al.*

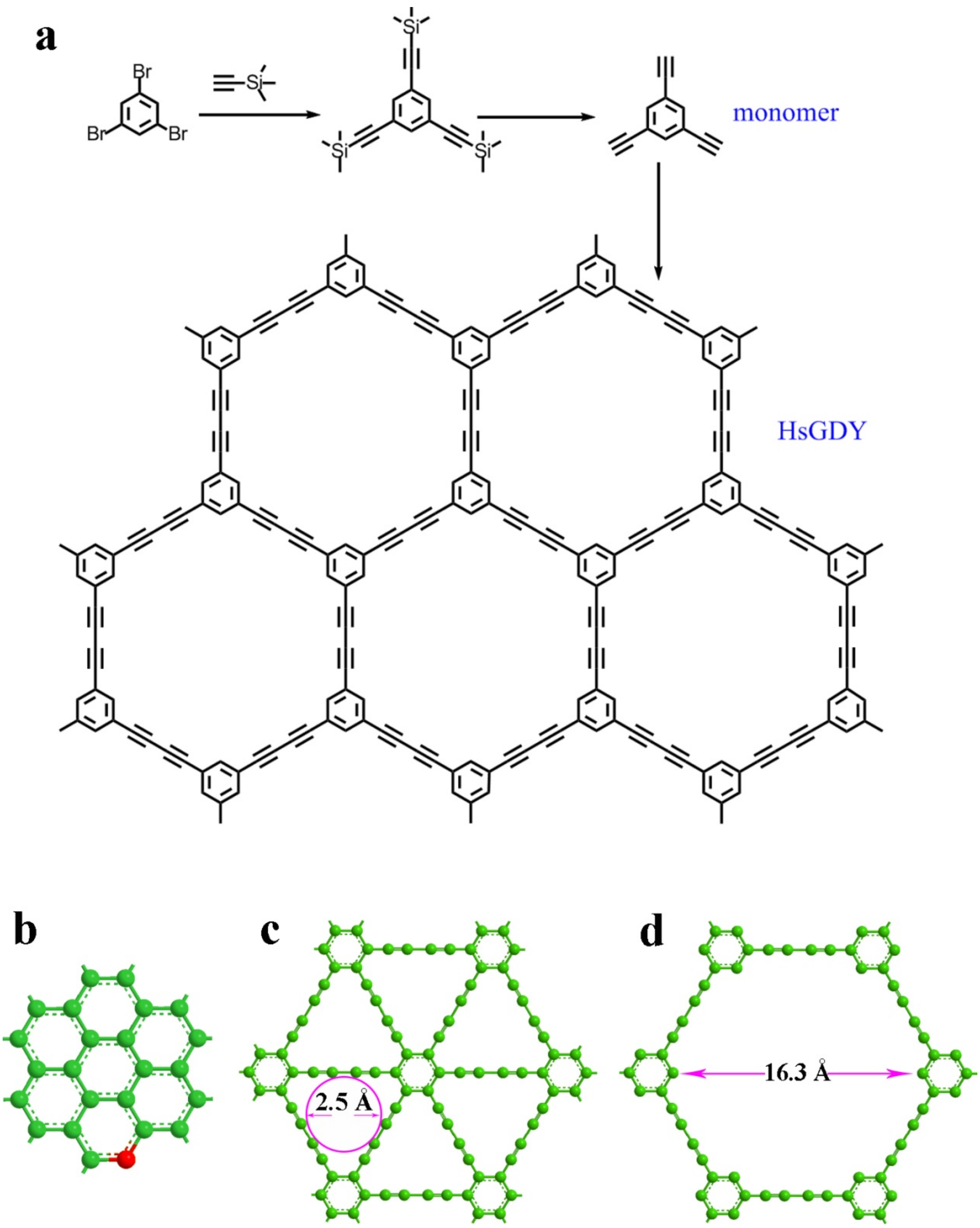

Supplementary Figure 1. a, The synthetic route of HsGDY. **b,**A fragment of pyridinic N doped graphene. pyridinic N locates at the edge of graphene. **c,** A fragment of GDY. The diameter of large pore is ca. 2.5 Å in GDY. **d,** A fragment of HsGDY. The diameter of large pore is ca. 16.3 Å in HsGDY.

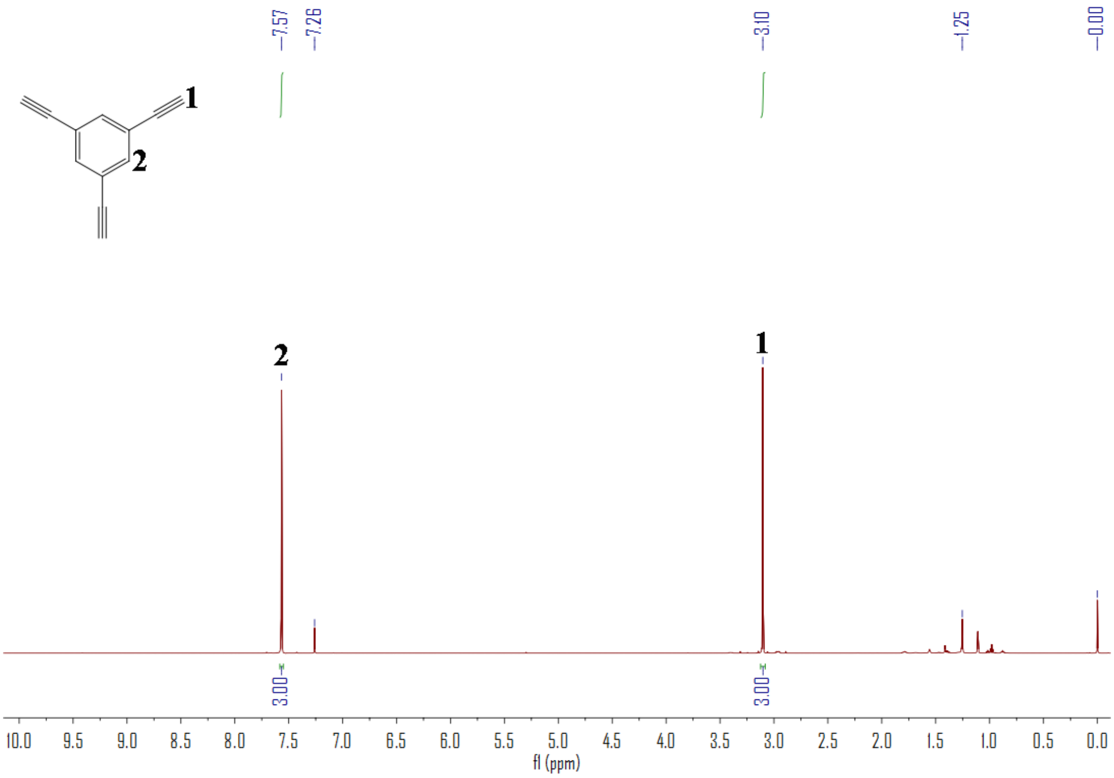

Supplementary Figure 2. ^{1}H NMR spectrum of Triethynylbenzene, the monomer of HsGDY.

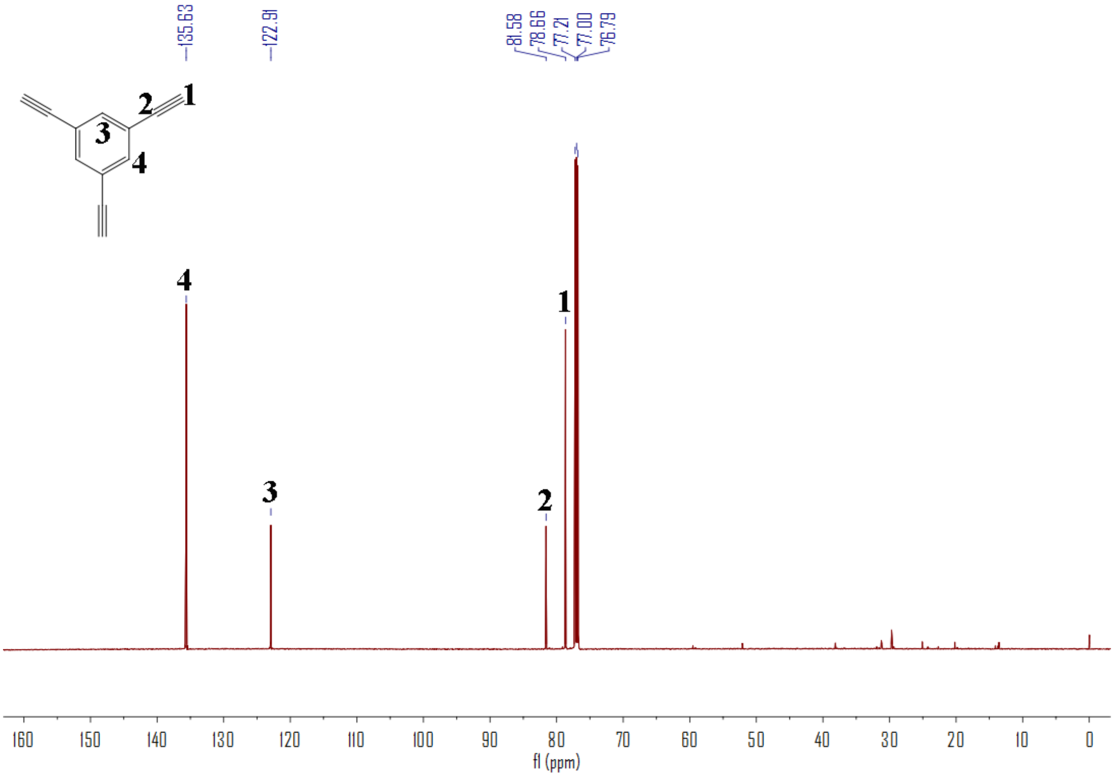

Supplementary Figure 3. ^{13}C NMR spectrum of Triethynylbenzene, the monomer of HsGDY.

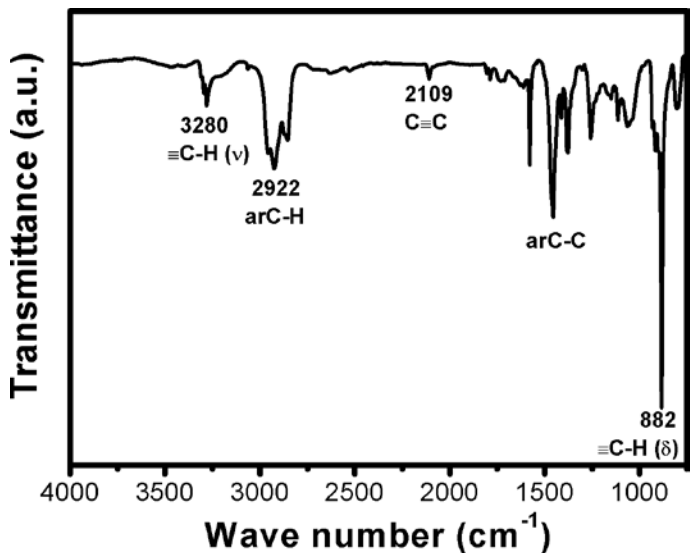

Supplementary Figure 4. FT-IR spectra for triethynylbenzene, the monomer of HsGDY.

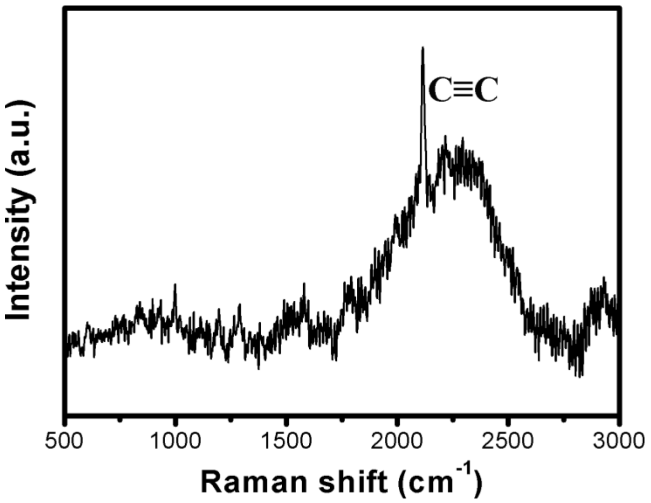

Supplementary Figure 5. Raman spectrum for triethynylbenzene, the monomer of HsGDY.

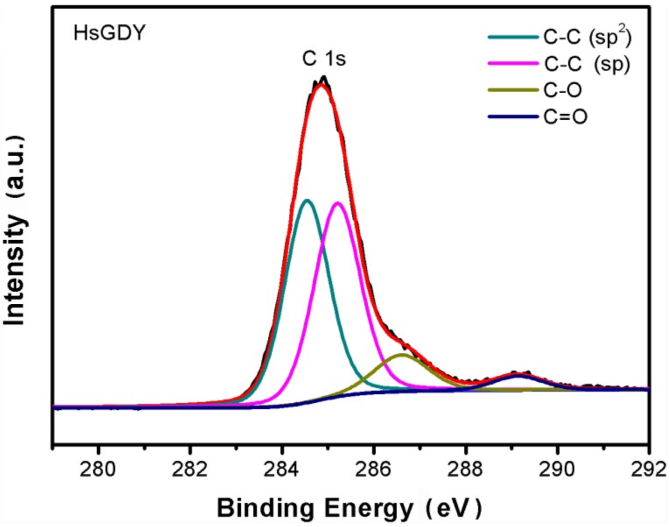

Supplementary Figure 6. C 1s region of XPS spectra for HsGDY.

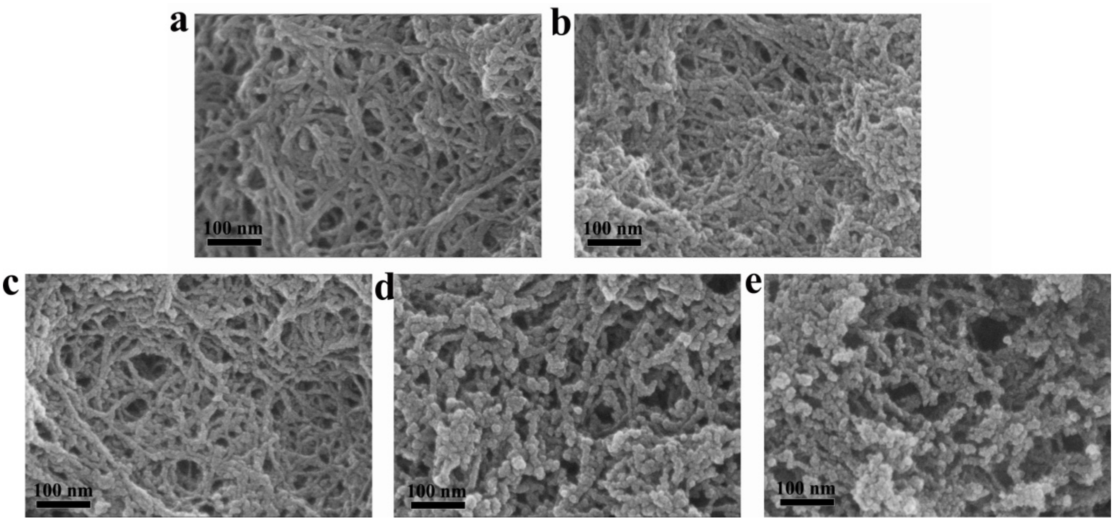

Supplementary Figure 7. a,b,c,d,e, SEM images of (a) HsGDY, (b) N-HsGDY-700 °C, (c) N-HsGDY-800 °C, (d) N-HsGDY-900 °C and (e) N-HsGDY-1000 °C.

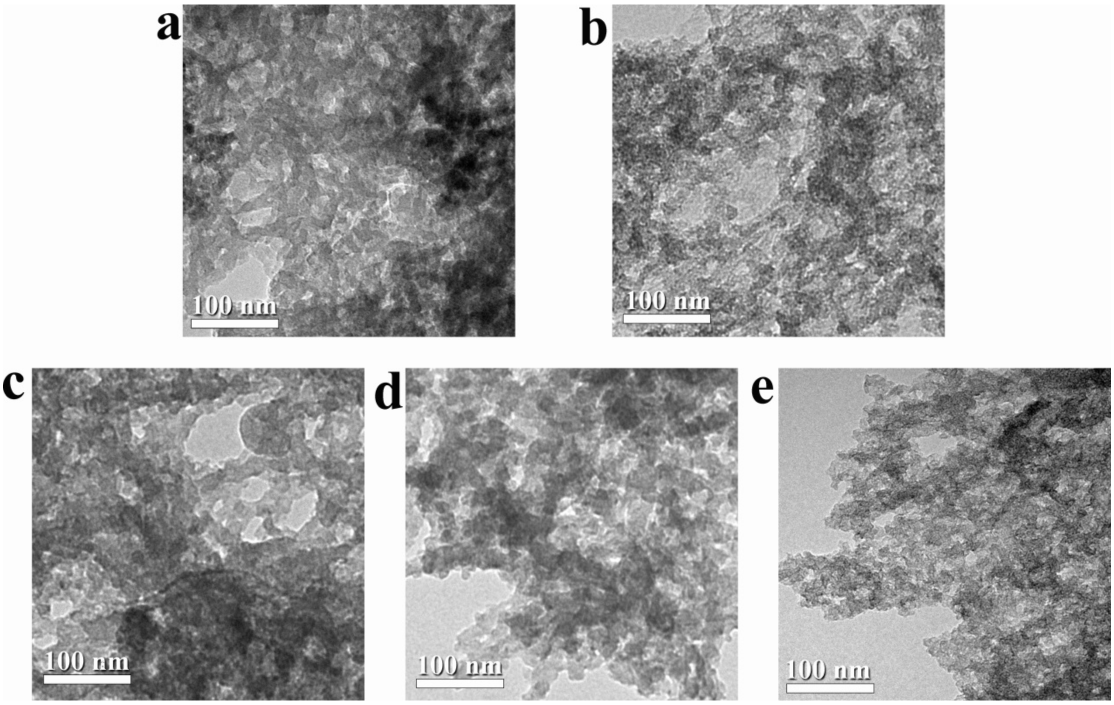

Supplementary Figure 8. a,b,c,d,e, TEM images of (a) HsGDY, (b) N-HsGDY-700 °C, (c) N-HsGDY-800 °C, (d) N-HsGDY-900 °C and (e) N-HsGDY-1000 °C.

Supplementary Note 1

The inductively coupled plasma was used to determine the content of Cu in the samples. It indicated that only trace amounts of Cu existed in both the N-HsGDY-900 °C and HsGDY (0.017% for N-HsGDY-900 °C, 0.014% for HsGDY).

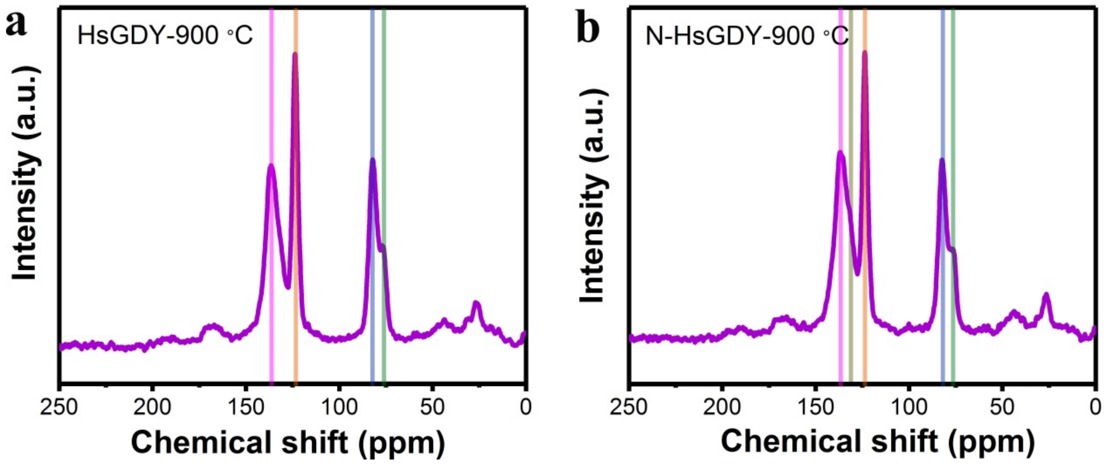

Supplementary Figure 9. ^{13}C solid-state NMR spectrum of HsGDY-900 ºC (a) and N-HsGDY-900 ºC (b).

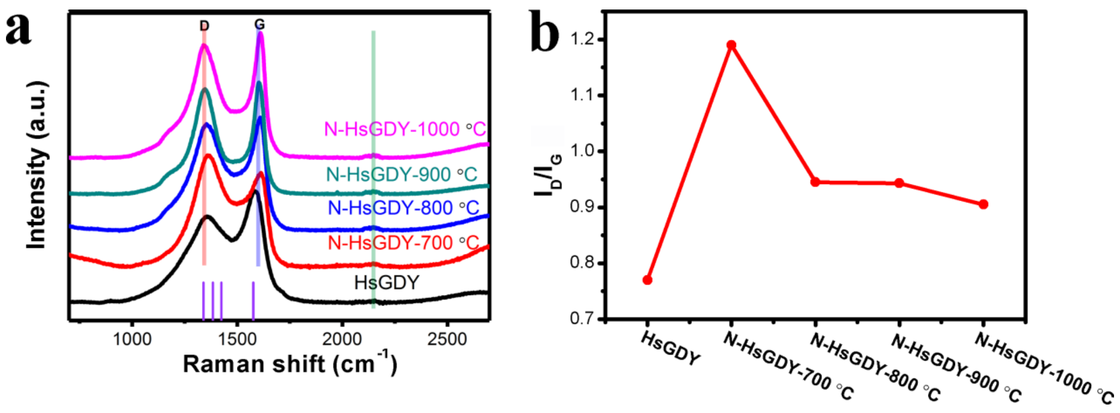

Supplementary Figure 10. a, Raman spectra of all the catalysts. The violet vertical curves represent the simulated peak positions of HsGDY calculated according to density functional theory. **b,** the change of I_D/I_G ratios of all the catalysts.

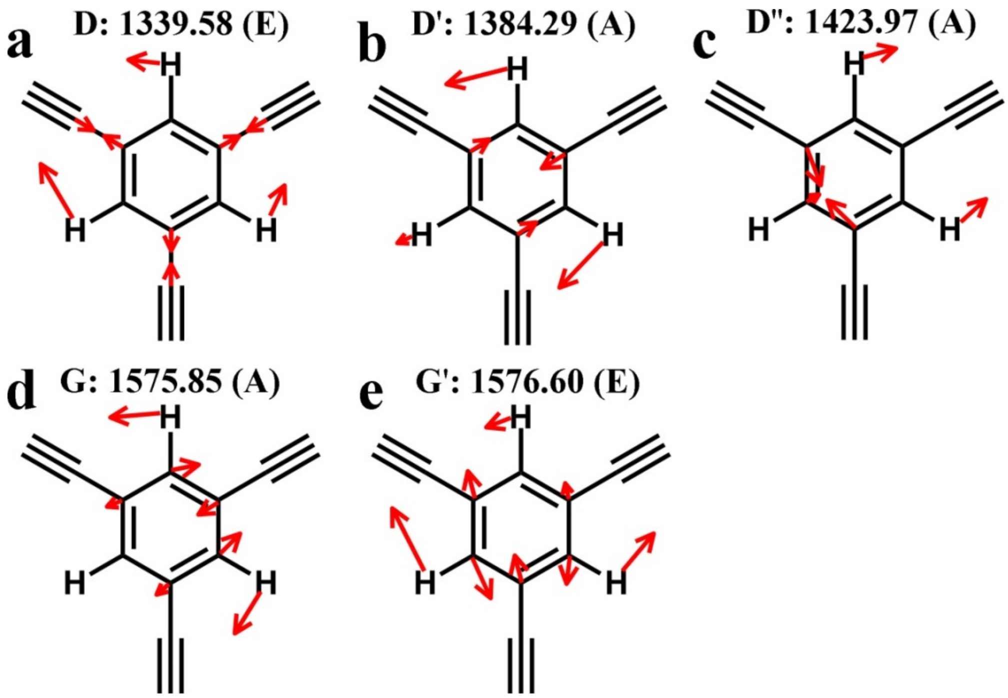

Supplementary Figure 11. Atomic motions of intense Raman-active modes, in which the red arrows show the motion directions of the main contributors.

Supplementary Note 2

To explain the high D peak of HsGDY, the Raman spectrum of D and G regions was simulated at B3LYP/6-311g** level by Gaussian 09 software. The results are shown in Supplementary Fig. 10a (the violet vertical curves) and 11. It can be seen that the high D peak of HsGDY are derived from the H atoms linked to aromatic ring. These H atoms can be considered as “defects”, compared to the total carbon conjugate materials.

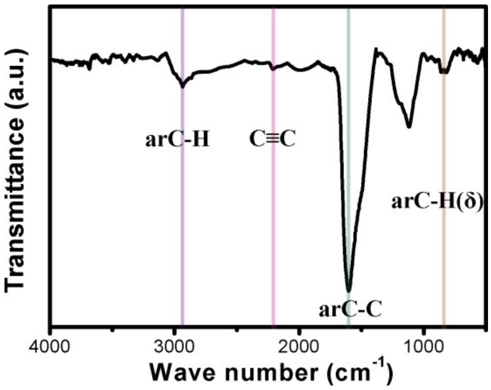

Supplementary Figure 12. FT-IR spectra of the N-HsGDY-900 °C.

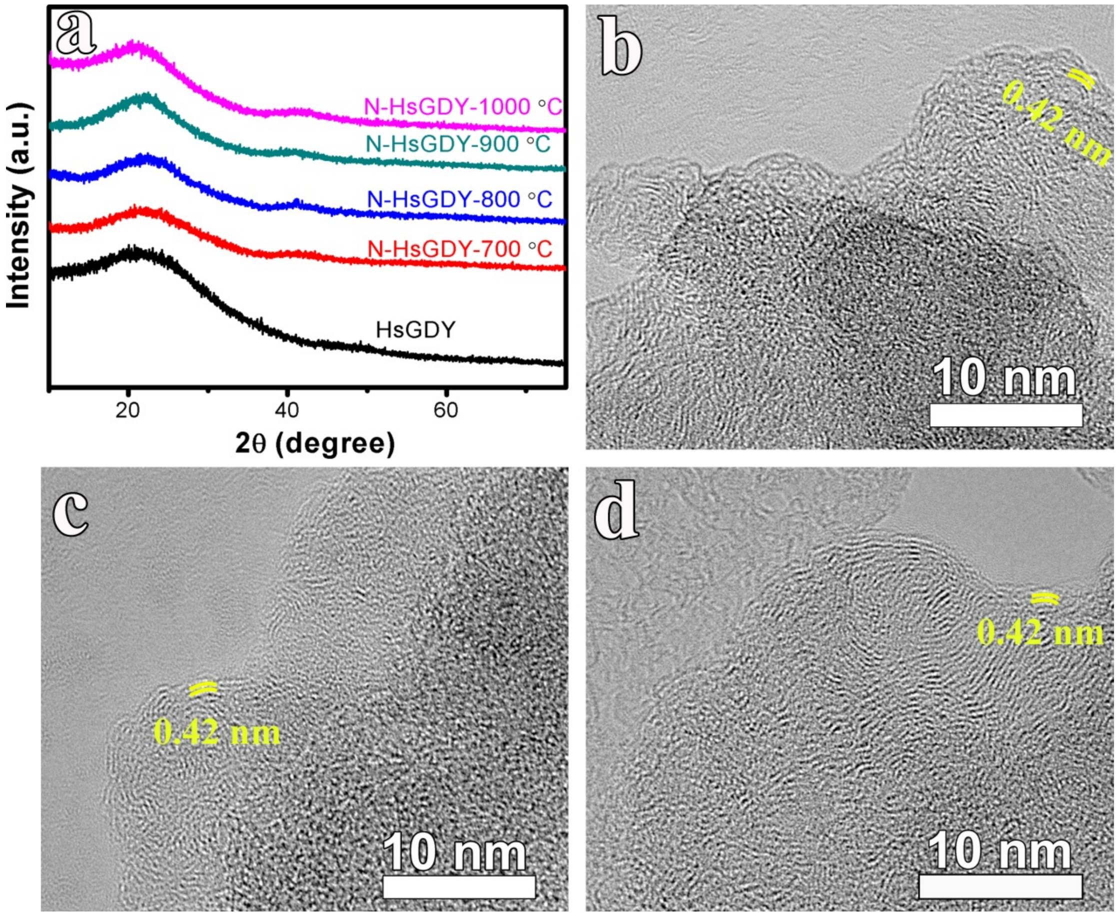

Supplementary Figure 13. a, X-ray diffraction (XRD) patterns for all the catalysts. **b,c,d,** HRTEM images for HsGDY (b), HsGDY-900 °C (c) and N-HsGDY-900 °C (d).

Supplementary Note 3

As shown in the HRTEM images (Supplementary Fig. 13), interlayer spacing stripes are remained for HsGDY-900 °C and N-HsGDY-900 ºC, similar to HsGDY. It indicates that the HsGDY-900 °C and N-HsGDY-900 ºC has layered structure with interlayer space of 0.42 nm, consistent with HsGDY.

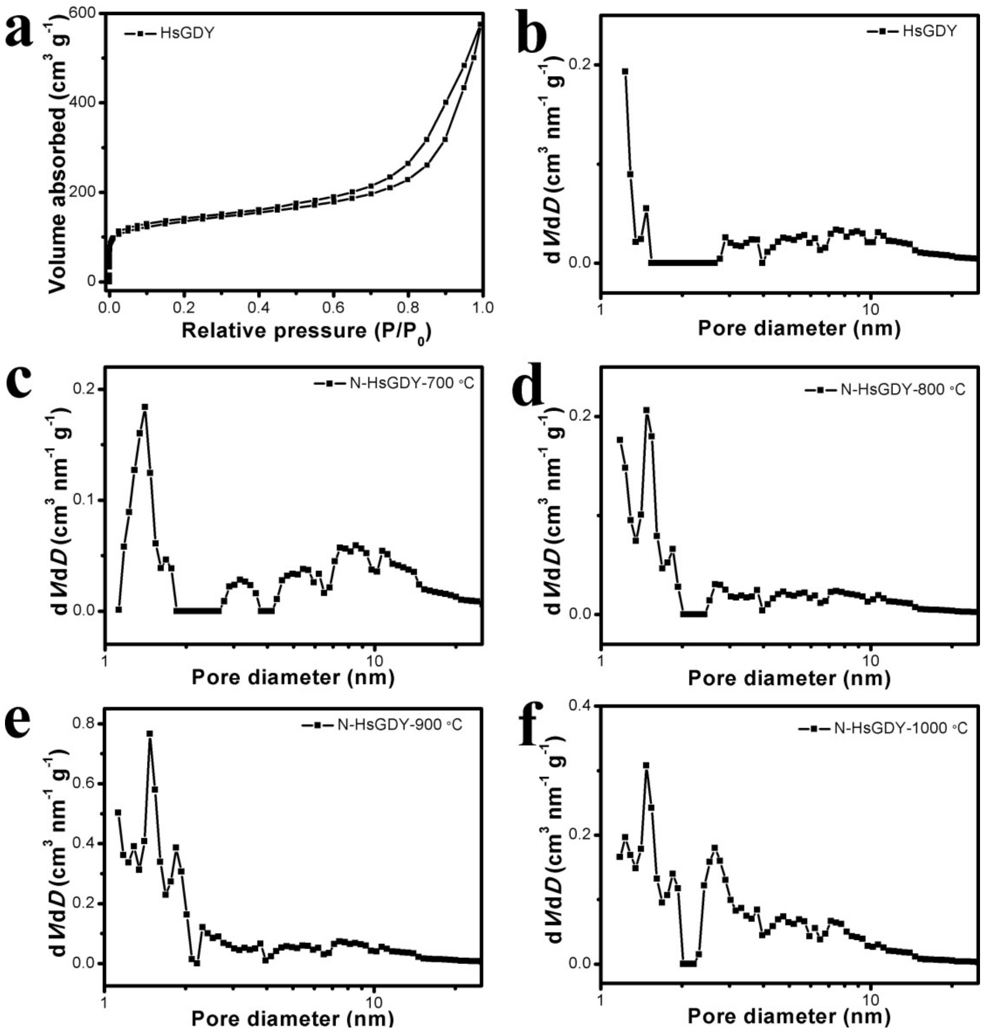

Supplementary Figure 14. a, N_2-adsorption/desorption isotherms for HsGDY. **b,c,d,e,** Pore-size distribution curves for HsGDY (b), N-HsGDY-700 °C (c), N-HsGDY-800 °C (d), N-HsGDY-900 °C (e) and N-HsGDY-1000 °C (f) calculated from the corresponding isotherms by the DFT method.

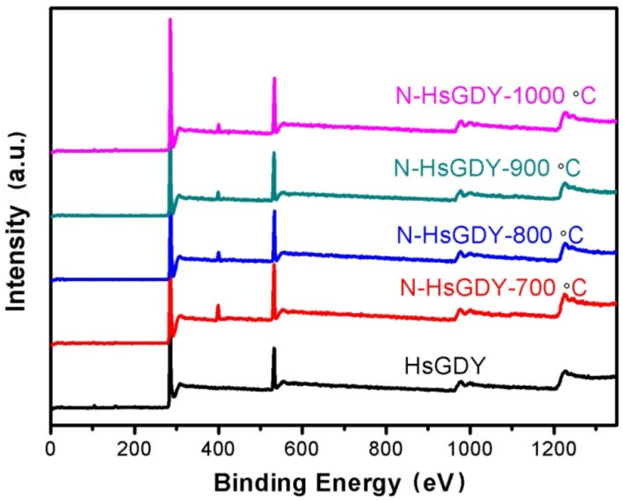

Supplementary Figure 15. The survey of XPS spectrum for all the catalysts.

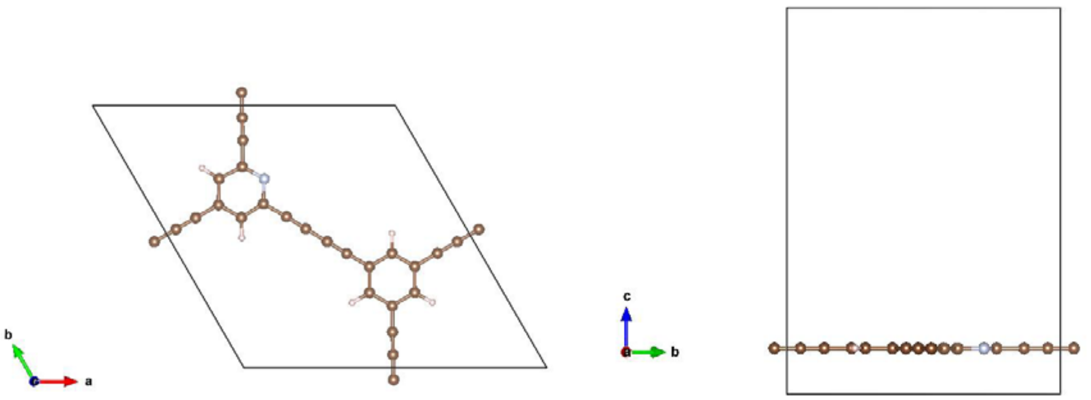

Supplementary Figure 16. The ball-and-stick model of a single-layer H-GDY of the c-axis view (Left panel) and the a-axis view (Right panel).

Supplementary Note 4

We calculated the theoretical K-edge XANES spectra of the pyridinic N site in N-HsGDY by density functional theory (DFT)[1, 2] implemented in Wien2k package[3, 4]. For a better agreement between the experimental and calculated XANES spectra, the core-hole approach with the main contribution from the electrical dipole transition is considered in the electronic structure computations. The electronic structures of H-GDY have been calculated with the full-potential linearized augmented plane wave (FLAPW) method. The exchange-correlation interaction is described by generalized gradient approximation (GGA) with the Perdew-Burke-Ernzerhof (PBE) functional[5]. The H-GDY geometry is shown in Supplementary Fig. 16, where its periodic alignment in the a-b plane is a hexagonal array with large vacuum distance of 20 Å in the c-axis. The values of the lattice parameters a and b are 16.32490 Å. The valence basis cutoff was set to -6 eV and the RK_{max}=8.0 was selected for a more accurate plane wave basis set. The total energy values were converged with accuracy to 1 meV for the 10×10×1 kpoint mesh. The N 1s core electron is excited to the valence electron for the core-hole approach and the theoretical K-edge XANES spectra of the pyridinic N site was obtained.

The enthalpy changes of N substituting C in HsGDY and graphene were calculated using density fuctional theory (DFT) with Gaussian 09 programs. The molecular structures were optimized using hybrid density functional method B3LYP with the 6-311(d,p) basis set.

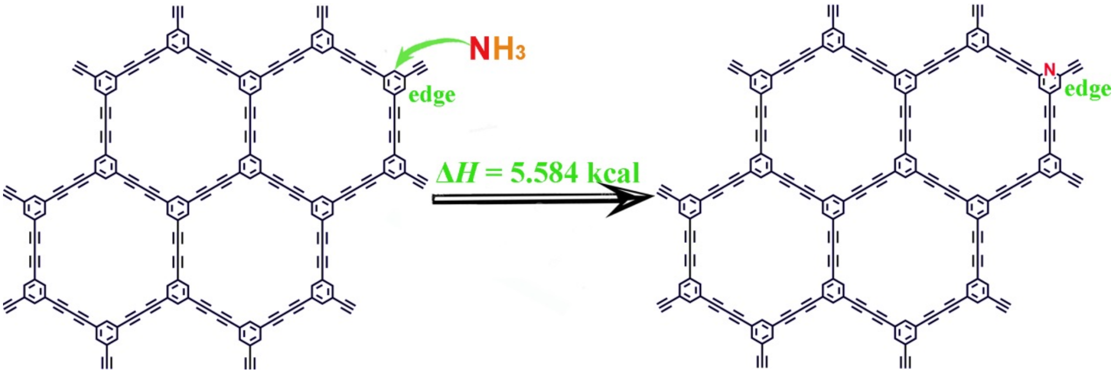

Supplementary Figure 17. Reaction equations and enthalpy changes of pyridinic N substituting C at the edge of HsGDY. The benzene ring at the edge of HsGDY was linked with alkynyl.

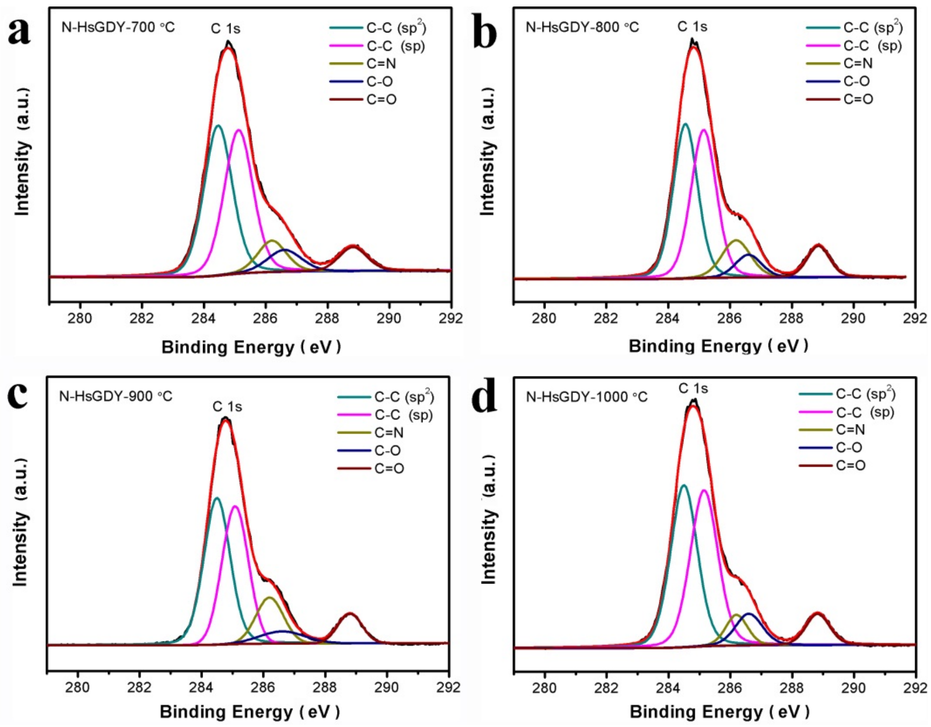

Supplementary Figure 18. a,b,c,d, C 1s region XPS spectra of all the N-HsGDY catalysts.

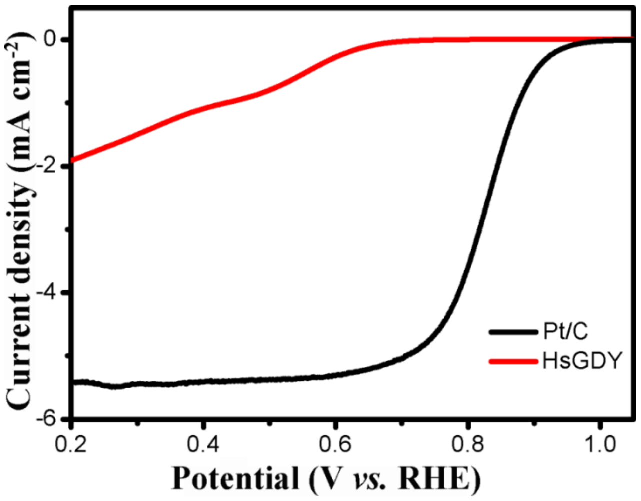

Supplementary Figure 19. Linear sweep voltammetry (LSV) curves of commercial Pt/C and HsGDY catalysts recorded in O_2-saturated 0.1 M KOH at 1600 rpm.

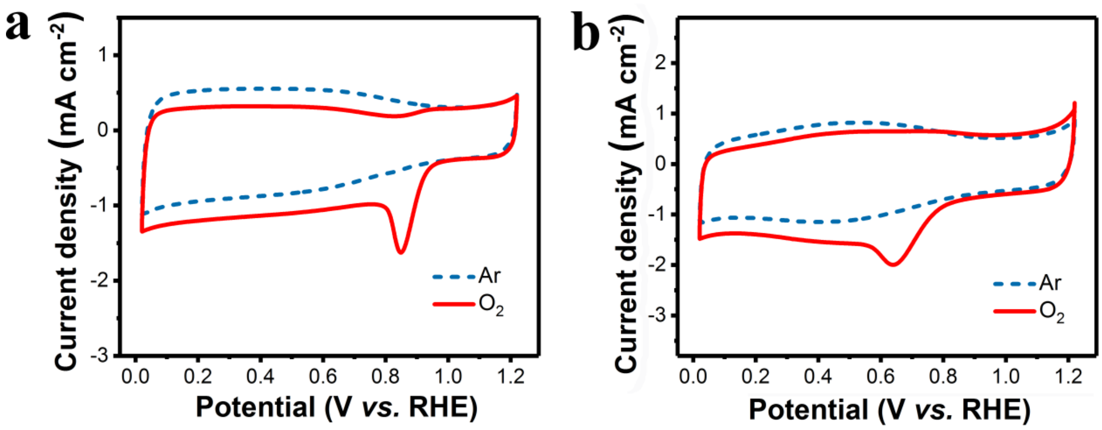

Supplementary Figure 20. a,b, CVs of the N-HsGDY-900 °C in O_2-saturated (solid) or Ar-saturated (dotted) 0.1 M KOH (a) and 0.1 M $HClO_4$ (b).

Supplementary Note 5

The cyclic voltammetry (CV) measurements of N-HsGDY-900 °C were conducted in a three-electrode electrochemical cell in both O_2-saturated or Ar-saturated 0.1 M KOH and 0.1 M $HClO_4$ as shown in Supplementary Fig. 20. An oxygen reduction peak at ~0.85 V (vs. RHE) or ~0.64 V (vs. RHE) can be observed in O_2-saturated KOH or 0.1 M $HClO_4$, respectively, which is not shown in Ar-saturated electrolyte. It indicates that N-HsGDY-900 °C holds high activity for ORR.

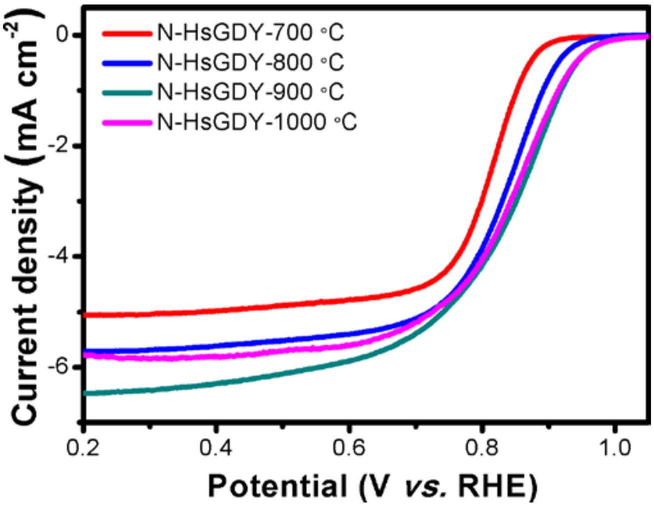

Supplementary Figure 21. Linear sweep voltammetry (LSV) curves of the N-HsGDY catalysts recorded in O_2-saturated 0.1 M KOH at 1600rpm, scan rate of 5 mV s^{-1}.

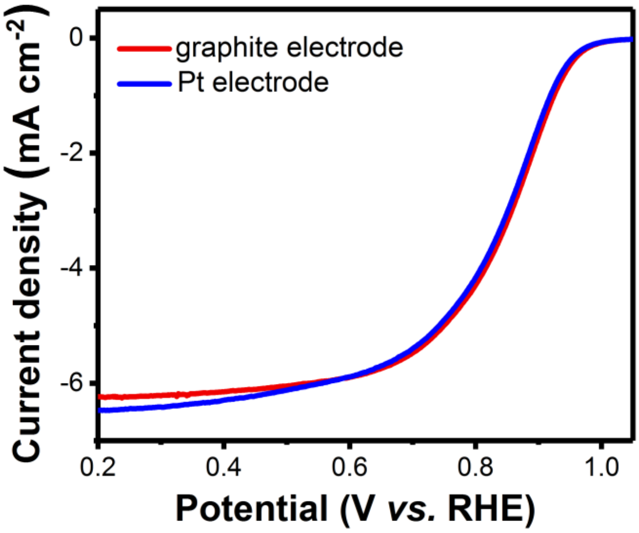

Supplementary Figure 22. LSV curves of N-HsGDY-900 °C tested with graphite rod or platinum plate as counter electrode in 0.1 M KOH solution.

Supplementary Note 6

To exclude the possible contamination of Pt counter electrode to the catalysts, LSV test of N-HsGDY-900 °C was conducted with a graphite rod as counter electrode as shown in the Supplementary Fig. 22. It can be seen that the Pt-contamination issue has little influence on the test.

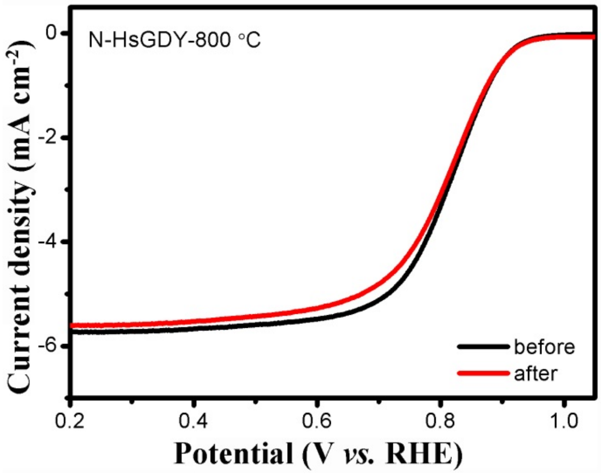

Supplementary Figure 23. Durability tests of the N-HsGDY-800 °C catalyst before and after 5000 cycles in O_2-saturated 0.1 M KOH.

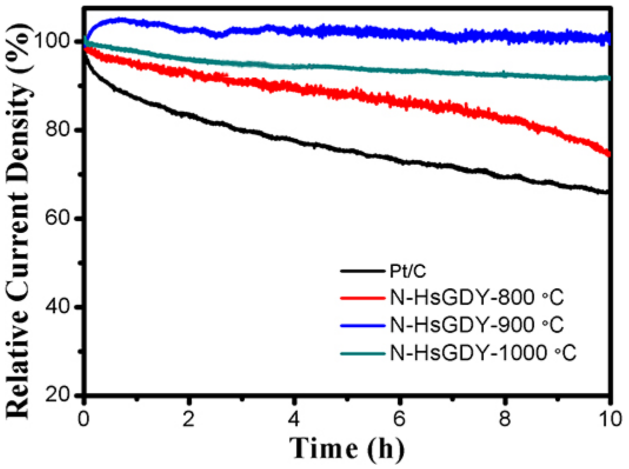

Supplementary Figure 24. Chronoamperometric measurements for Pt/C, N-HsGDY-800 °C, N-HsGDY-900 °C and N-HsGDY-1000 °C in O_2-saturated 0.1 M KOH solution.

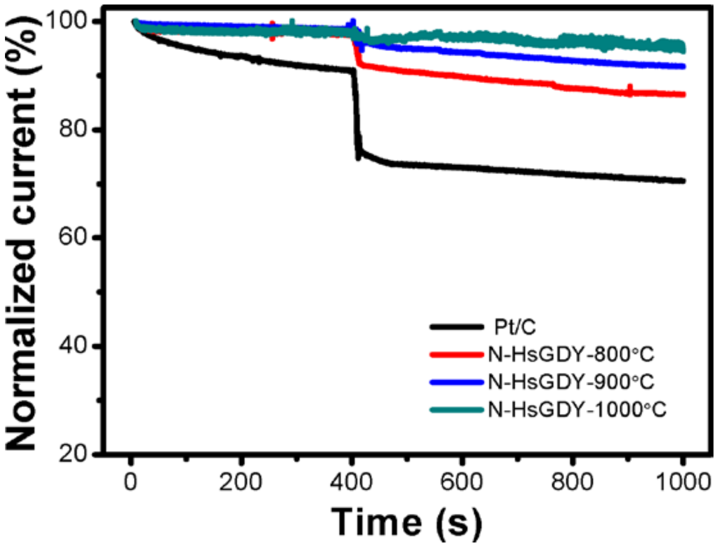

Supplementary Figure 25. Methanol tolerance experiments with methanol injected at 400 s. Chronoamperometric measurements were conducted at 0.8 V vs. RHE in O_2-saturated 0.1 M KOH with rotating rate of 1600rpm.

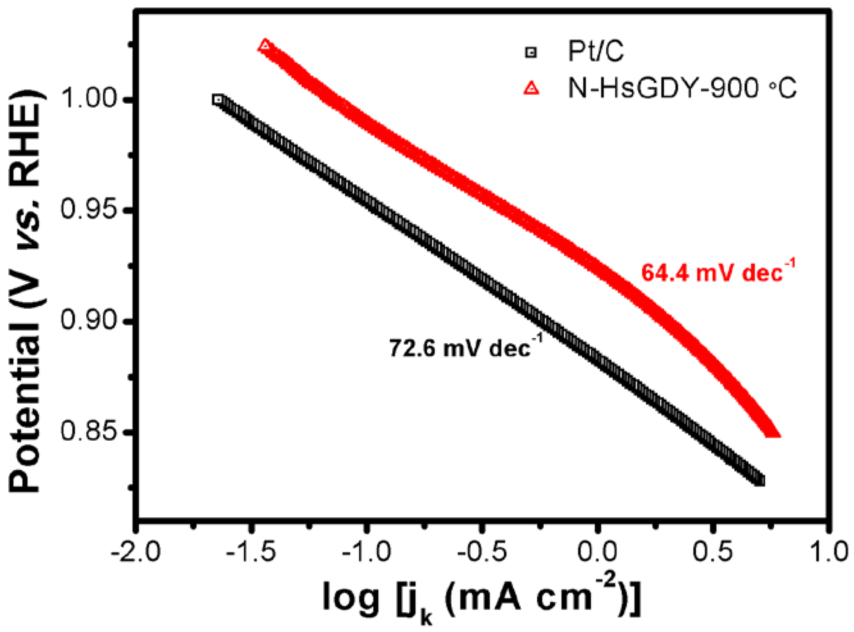

Supplementary Figure 26. Tafel plots for Pt/C and N-HsGDY-900 °C transformed from the LSV tests in 0.1 M KOH solution.

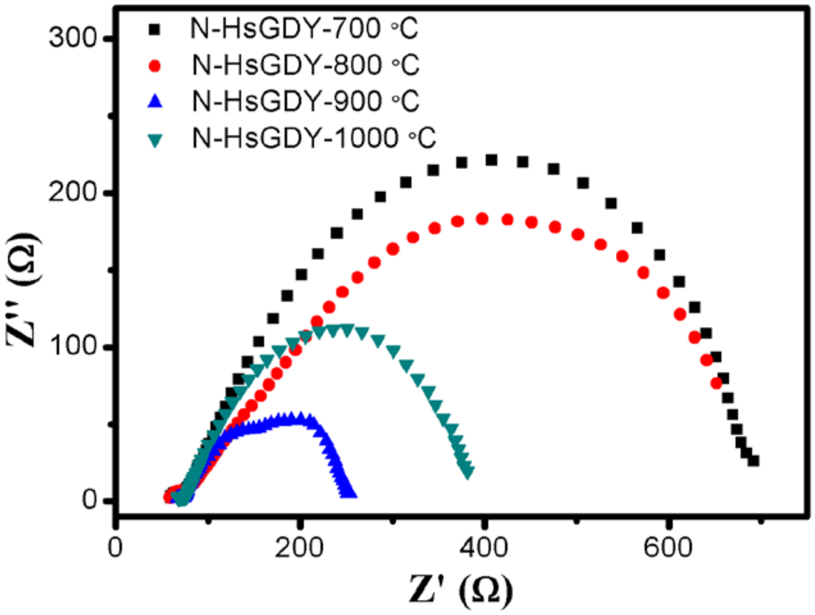

Supplementary Figure 27. Electrochemical impedance spectroscopy of N-HsGDY-700 °C, N-HsGDY-800 °C, N-HsGDY-900 °C and N-HsGDY-1000 °C at 0.85 V (*vs.* RHE) in O_2-saturated 0.1 M KOH at rotating rate of 1600 rpm.

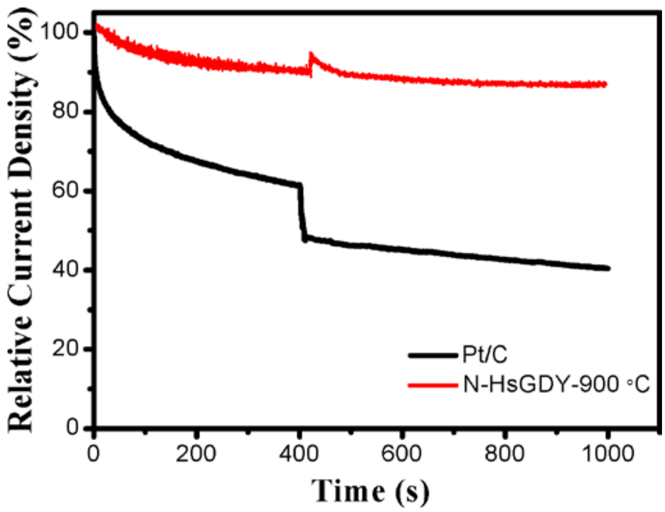

Supplementary Figure 28. Methanol tolerance experiments with methanol injected at 400 s. Chronoamperometric measurements were conducted at 0.65 V vs. RHE in O_2-saturated 0.1 M $HClO_4$ with rotating rate of 1600 rpm.

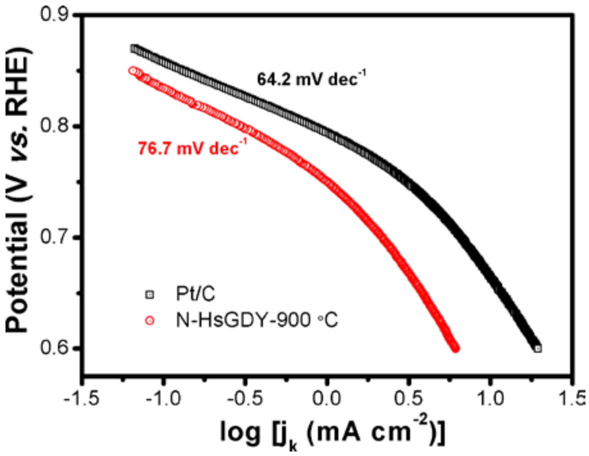

Supplementary Figure 29. Tafel plots for Pt/C, and N-HsGDY-900 °C transformed from the LSV tests in 0.1 M $HClO_4$ solution.

Supplementary Note 7: Calculation methods and models

The DFT calculation was performed using the Vienna Ab initio Simulation Package (VASP) code[6, 7], with exchange correlation effects being described by the Perdew−Burke−Ernzerhof (PBE) version of the generalized gradient approximation (GGA)[5, 8]. A plane-wave basis set with kinetic-energy cutoff of 500 eV has been used. The convergence criterion for the electronic structure iteration was set to be 10^{-4} eV, and that for geometry optimizations was set to be 0.01 eV/Å on force. To better describe the dispersion interaction within water adsorption systems, vdW correction was considered by adopting the Grimme's D2 scheme[9]. The optimized lattice parameters for HsGDY and graphene were 16.55 Å × 16.46 Å and 2.48 Å × 2.48 Å, respectively. N-doped HsGDY used a unit cell of HsGDY. The constructed supercell for N-graphene contained a 5 × 4 unit cells in the box with the size of 5 × 8 unit cells. All of the single layer structures were separated by a distance of 20 Å between two layers in the *z* direction. A Monkhorst-Pack 3 × 3 × 1 k-point grid was used for N-HsGDY and a 3 × 1 × 1 k-point grid was used for N-graphene.

The ORR and reaction pathway on the N-doped HsGDY and graphene models were calculated. The computational hydrogen electrode was utilized to obtain free energies for each state as done in ref [10]. In alkaline media, the four-electron ORR pathway could be summarized by the following elementary steps[11, 12]:

$$O_2(g) + 2H_2O(l) + 4e^- + * \rightarrow OOH^* + OH^- + H_2O(l) + 3e^- \quad \text{(S1a)}$$

$$OOH^* + OH^- + H_2O(l) + 3e^- \rightarrow O^* + 2OH^- + H_2O(l) + 2e^- \quad \text{(S1b)}$$

$$O^* + 2OH^- + H_2O(l) + 2e^- \rightarrow OH^* + 3OH^- + e^- \quad \text{(S1c)}$$

$$OH^* + 3OH^- + e^- \rightarrow 4OH^- + * \quad \text{(S1d)}$$

The free energies of reactants and each intermediate state at an applied electrode potential U were calculated as follows: $G(\mathrm{U}) = \Delta E + \Delta \mathrm{ZPE} - \mathrm{T}\Delta S - neU$, where n is the electron number of such state and ΔE represents the change in enthalpy which is considered from the DFT total energy value, ΔZPE represents the change in zero point energy and ΔS represents the change in entropy. Since it is difficult to obtain the exact free energy of OOH, O, and OH radicals in the electrolyte solution, the adsorption free energy ΔG_{OOH*}, ΔG_{O*}, and ΔG_{OH*} are used in the calculations. At equilibrium potential U^0, the free energy change of four steps could be

obtained as follow:

$$\Delta G_A(U^0) = G_{OOH*} + G_{H_2O(l)} - G_* - 3G_{OH^-} - 3eU^0 \quad \text{(S2a)}$$

$$\Delta G_B(U^0) = G_{O*} - G_{OOH*} + G_{OH^-} - eU^0 \quad \text{(S2b)}$$

$$\Delta G_C(U^0) = G_{OH*} - G_{H_2O(l)} - G_{O*} + G_{OH^-} - eU^0 \quad \text{(S2c)}$$

$$\Delta G_D(U^0) = -G_{OH*} + G_* + G_{OH^-} + eU^0 \quad \text{(S2d)}$$

The equilibrium potential U^0 for ORR was determined to be 0.455 V *vs* NHE alkaline media and 1.229 V *vs* NHE acidic media where the reactant and product are at the same energy level. The free energy of $H_2O(l)$ is derived as $G_{H2O(l)} = G_{H2O(g)} + RT \times \ln(p/p0)$ since only $G_{H2O(g)}$ can be directly obtained by DFT calculations, where R is the ideal gas constant, T = 298.15K, p = 0.035 bar, and $p0$ = 1 bar. The free energy of $O_2(g)$ has been estimated as $G_{O2(g)} = 2G_{H2O(l)} - 2G_{H2} - 4.92$ eV. The free energy of OH^- was derived as $G_{OH-} = G_{H2O(l)} - G_{H+}$, where $G_{H+} = 1/2G_{H2} - k_BT\ln 10 \times pH$. More information about the free energies of reactions can be found in the ref 12.

In acidic media, the four-electron ORR pathway could be summarized by the following elementary steps:

$$O_2(g) + 4H^+ + 4e^- + * \rightarrow OOH* + 3H^+ + 3e^- \quad \text{(S3a)}$$

$$OOH* + 3H^+ + 3e^- \rightarrow O* + H_2O(l) + 2H^+ + 2e^- \quad \text{(S3b)}$$

$$O* + H_2O(l) + 2H^+ + 2e^- \rightarrow OH* + H_2O(l) + H^+ + e^- \quad \text{(S3c)}$$

$$OH* + H_2O(l) + H^+ + e^- \rightarrow 2H_2O(l) + * \quad \text{(S3d)}$$

Supplementary Table 1 below shows the adopted values of entropy and zero point energy for the adsorption and gas phase states of molecules[10, 11].

Supplementary Table 1. Entropy and zero point energy for the adsorption and gas phase states of molecules.

	TS (eV)	ZPE (eV)
H_2O	0.67	0.56
H_2	0.41	0.27
OOH*	0	0.35
OH*	0.01	0.31
O*	0	0.05

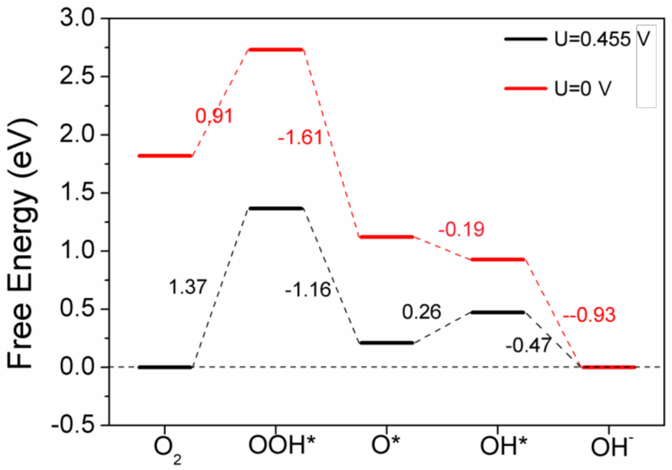

Supplementary Figure 30. A free energy diagram of ORR on pyridinic N doped HsGDY in which aromatic carbon are active sites. The atomic configurations for ORR intermediate states are shown in supplementary Fig. 31b.

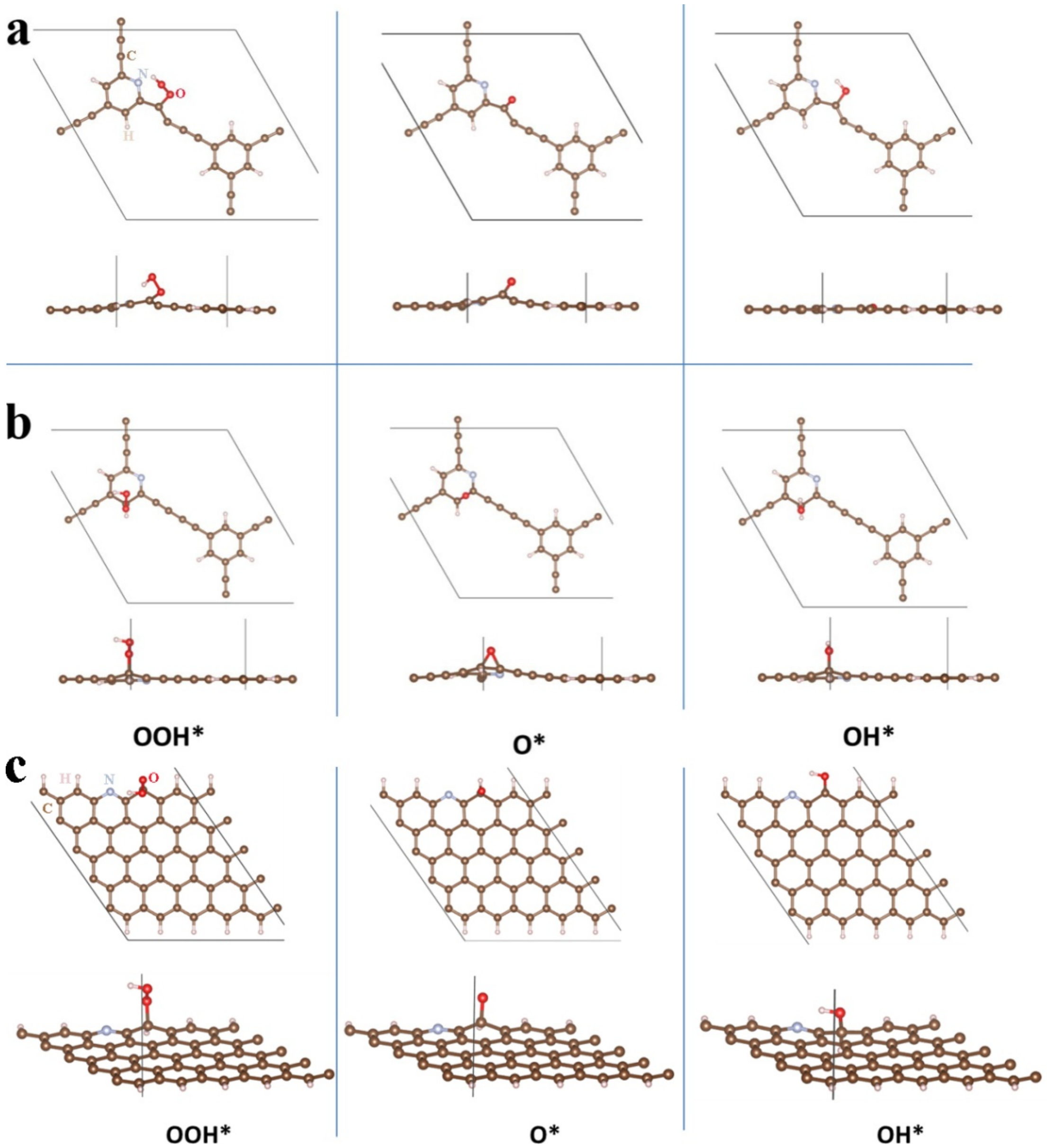

Supplementary Figure 31. a, The optimized atomic configurations of ORR intermediate states with a acetylenic carbon as active site in alkaline media, corresponding to the free energy diagram in Fig. 7a. **b,** The atomic configurations of ORR intermediate states with a acetylenic carbon as active site in alkaline media, corresponding to the free energy diagram in Supplementary Fig. 30. **c,** The optimized atomic configuration of ORR intermediate states for pyridinic N doped graphene in alkaline media, corresponding to the free energy diagram in Fig. 7b.

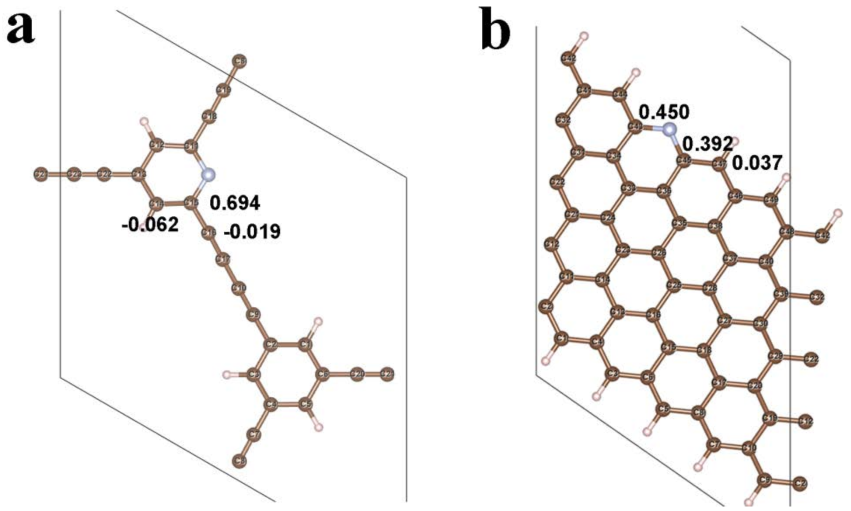

Supplementary Figure 32. a,b, The charge distribution of pyridinic N doped HsGDY (a) and pyridinic N doped graphene (b).

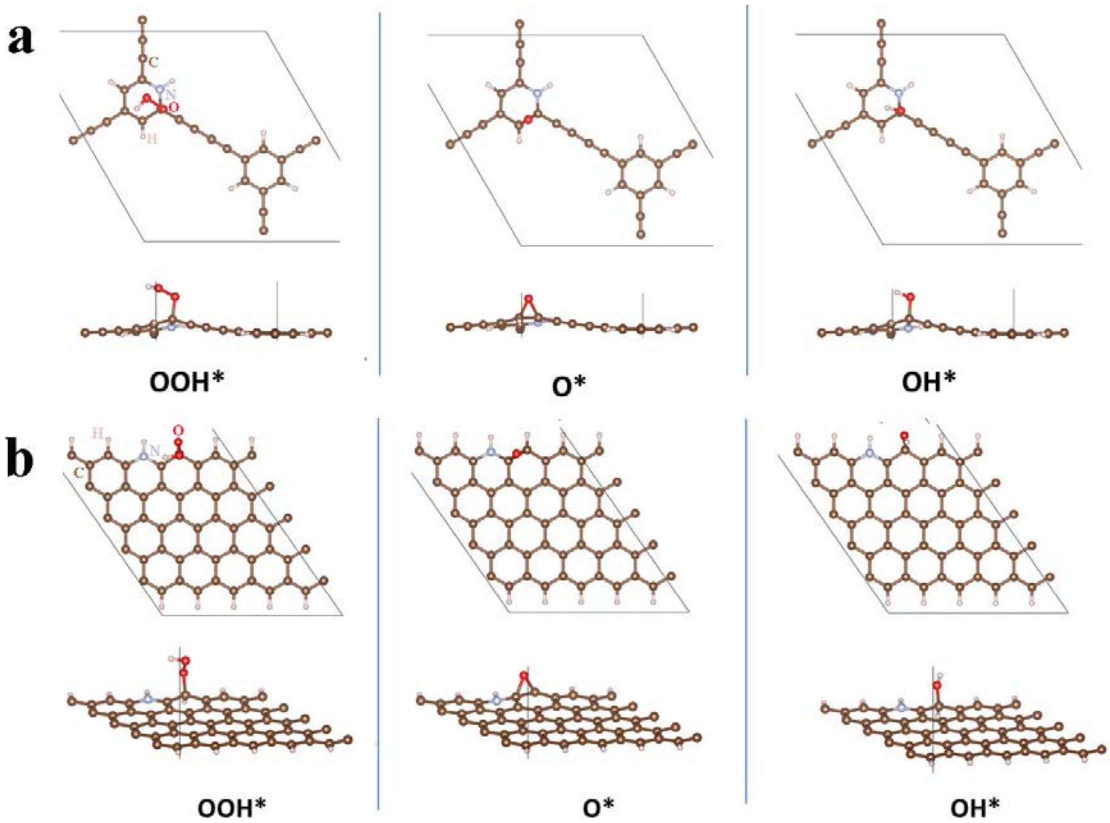

Supplementary Figure 33. a, The optimized atomic configurations of ORR intermediate states for pyridinic N doped HsGDY in acidic media. **b,** The optimized atomic configurations of ORR intermediate states for pyridinic N doped graphene in acidic media.

Supplementary Table 2. Catalytic activities for ORR of metal-free catalysts in this work and recently reported materials. All the catalysts were tested in 0.1 M KOH solution.

Electrocatalysts	Onset potential (V *vs.* RHE)	Half-wave potential (V *vs.* RHE)	Limiting-current density (mA cm^{-2})	References
**N-HsGDY-900 °C**	**1.02**	**0.85**	**6.5**	**This work.**
N-Carbon Nanotube Arrays	0.97	0.84	5.6	[13]*Science* **2009,** *323* (5915), 760-764.
N-Porous Carbon Nanosheets	0.9	0.77	5.8	[14] *Adv. Mater.* **2016,** *28*, 5080
N-porous carbon	0.98	0.88	5.5	[15]*Adv. Energy Mater*. **2016,** *6* (8), 1502389.
N-Nanoporous Carbon Fiber Film	0.97	0.82	4.7	[16]*Adv. Mater.* **2016,** *28* (15), 3000-3006.
N-Porous Carbon Superstructures	0.93	0.84	5.5	[17]*Adv. Mater.* **2016,** *28* (10), 1981-1987.
N-graphene mesh	0.89	0.77	6.1	[18]*Adv. Mater.* **2016,** *28* (32), 6845-6851.
N superdoped graphene	0.97	0.79	4.7	[19] *Nat Commun* **2016,** *7*, 10921.
N-Meso-Microporous Carbon Frameworks	0.88	0.82	4.7	[20] *Adv. Funct. Mater.* **2016,** *26* (45), 8334-8344.
Pyridinic N Dominated Graphene Aerogels	0.89	0.75	4.6	[21] *Adv. Funct. Mater.* **2016,** *26* (31), 5708-5717.
N-hierarchical porous carbon	0.94	0.85	5.4	[22]*ACS Nano* **2016,** *10* (4), 4364-4371.
N-hierarchically porous carbons	0.92	0.87	5.8	[23]*Nat. Commun.* **2014,** *5*, 4973.
N-mesoporous carbon spheres	0.89	0.71	5.6	[24]*Angew. Chem. Int. Ed.* **2015,** *54* (2), 588-593.
B,N,S-1D porous carbons	0.92	0.75	4.3	[25] *Adv. Funct. Mater.* **2016,** *26* (45), 8255-8265
N-ordered mesoporous carbon/graphene framework	0.93	0.82	5.0	[26] *Nano Energy* **2016,** *30*, 503-510.
N-Doped Mesoporous Carbon	1.02	0.90	5.8	[27]*Adv. Funct. Mater.* **2017,** *27* (14), 1606190.
N, S-graphitic sheets	1.01	0.87	5.1	[28] *Adv. Mater.* **2017,** *29* (9), 1604942

P,N-graphene framework	0.94	0.84	5.6	[29] *Energy Environ. Sci.* **2017,** *10* (5), 1186-1195.
N,P-Carbon Network	0.98	0.82	5.0	[30] *Angew. Chem. Int. Ed.* **2016,** *55* (6), 2230-2234.
N,P-Carbon Nanotubes/Graphene hybrid nanospheres	0.94	0.82	5.5	[31] *Adv. Mater.* **2016,** *28* (23), 4606-4613.
S,N-Porous Carbon Nanosheets	0.86	0.74	5.0	[32] *Adv. Funct. Mater.* **2016,** *26* (32), 5893-5902.
N,P-mesoporous carbon	0.94	0.85	4.3	[33] *Nat Nano* **2015,** *10* (5), 444-452.
Te,P-porous carbon fiber	0.89	0.79	5.7	[34] *J. Am. Chem. Soc.* **2014,** *136* (41), 14385-14388.

Supplementary Table 3. Catalytic activities for ORR of metal-free catalysts in this work and recently reported materials, tested in acidic media.

Electrocatalysts	Media	Onset potential	Half-wave potential	Limiting-current density	References
**N-HsGDY-900°C**	**0.1 M $HClO_4$**	**0.86**	**0.64**	**4.7**	**This work.**
N-graphene mesh	0.1 M $HClO_4$	0.80	0.31	5.1	[18]*Adv. Mater.* **2016,** *28* (32), 6845-6851.
N-mesoporous Carbon	0.5 M H_2SO_4	0.82	0.70	5.8	[27]*Adv. Funct. Mater.* **2017,** *27* (14), 1606190.
N-mesoporous carbon	0.1 M $HClO_4$	0.8	0.5	4.5	[35]*J. Am. Chem. Soc.* **2011,** *133* (2), 206-209.
N-carbon speres	0.5 M H_2SO_4	0.65	0.42	5.5	[36]*Adv. Mater.* **2013,** *25* (7), 998-1003.
N-meso/micro porous carbon	0.5 M H_2SO_4	0.84	0.72	4.6	[23]*Nat. Commun.* **2014,** *5*, 4973.
N-Carbon Nanosheet	0.5 M H_2SO_4	0.75	0.57	5.0	[37]*Angew. Chem.* **2014,** *126* (6), 1596-1600.
N-nanoporous carbon nanosheets	0.5 M H_2SO_4	0.72	0.40	3.3	[38]*Energy Environ. Sci.* **2014,** *7* (12), 4095-4103.
N,P-Carbon Nanotubes/Graphene hybrid nanospheres	0.1 M $HClO_4$	0.90	0.68	5.7	[31] *Adv. Mater.* **2016,** *28* (23), 4606-4613.
N-Porous Carbon Superstructures	0.5 M H_2SO_4	-	0.66	5.3	[17]*Adv. Mater.* **2016,** *28* (10), 1981-1987.
N,P-mesoporous carbon	0.1 M $HClO_4$	0.82	0.62	5.6	[33]*Nat Nano* **2015,** *10* (5), 444-452.
S,N-Porous Carbon Nanosheets	0.5 M H_2SO_4	0.81	0.36	2.8	[32] *Adv. Funct. Mater.* **2016,** *26* (32), 5893-5902.
N,S-Graphene Nanosheets	0.5 M H_2SO_4	0.55	0.29	4.0	[39]*ChemCatChem* **2017,** *9* (6), 987-996.

Supplementary References

1. Hohenberg, P. & Kohn, W. Inhomogeneous electron gas. *Phys. Rev.* **136**, B864-B871 (1964).
2. Kohn, W. & Sham, L.J. Self-consistent equations including exchange and correlation effects. *Phys. Rev.* **140**, A1133-A1138 (1965).
3. Blaha, P., Schwarz, K., Sorantin, P. & Trickey, S.B. Full-potential, linearized augmented plane wave programs for crystalline systems. *Comput. Phys. Commun.* **59**, 399-415 (1990).
4. Blaha, P. & Schwarz, K. A full-potential LAPW study of structural and electronic properties of beryllium. *J. Phys. F: Met. Phys.* **17**, 899 (1987).
5. Perdew, J.P., Burke, K. & Ernzerhof, M. Generalized gradient approximation made simple. *Phys. Rev. Lett.* **77**, 3865-3868 (1996).
6. Kresse, G. & Furthmüller, J. Efficiency of ab-initio total energy calculations for metals and semiconductors using a plane-wave basis set. *Comput. Mater. Science* **6**, 15-50 (1996).
7. Kresse, G. & Hafner, J. Ab initio molecular dynamics for liquid metals. *Phys. Rev. B* **47**, 558-561 (1993).
8. Perdew, J.P., Burke, K. & Ernzerhof, M. Generalized gradient approximation made simple. *Phys. Rev. Lett.* **78**, 1396-1396 (1997).
9. Grimme, S. Semiempirical GGA-type density functional constructed with a long-range dispersion correction. *J. Comput. Chem.* **27**, 1787-1799 (2006).
10. Norskov, J.K. et al. Origin of the overpotential for oxygen reduction at a fuel-cell cathode. *J Phys. Chem. B* **108**, 17886-17892 (2004).
11. Zheng, Y. et al. Molecule-level g-C_3N_4 coordinated transition metals as a new class of electrocatalysts for oxygen electrode reactions. *J. Am. Chem. Soc.* **139**, 3336-3339 (2017).
12. Jiao, Y., Zheng, Y., Jaroniec, M. & Qiao, S.Z. Origin of the electrocatalytic oxygen reduction activity of graphene-based catalysts: a roadnnap to achieve the best performance. *J. Am. Chem. Soc.* **136**, 4394-4403 (2014).
13. Gong, K., Du, F., Xia, Z., Durstock, M. & Dai, L. Nitrogen-doped carbon nanotube arrays with high electrocatalytic activity for oxygen reduction. *Science* **323**, 760-764 (2009).
14. Yu, H. et al. Nitrogen-doped porous carbon nanosheets templated from g-C_3N_4 as metal-free electrocatalysts for efficient oxygen reduction reaction. *Adv. Mater.* **28**, 5080-5086 (2016).
15. Pampel, J. & Fellinger, T.-P. Opening of bottleneck pores for the improvement of nitrogen doped carbon electrocatalysts. *Adv. Energy Mater.* **6**, 1502389 (2016).
16. Liu, Q., Wang, Y., Dai, L. & Yao, J. Scalable fabrication of nanoporous carbon fiber films as bifunctional catalytic electrodes for flexible Zn-Air batteries. *Adv. Mater.* **28**, 3000-3006 (2016).
17. Xu, Z. et al. Nitrogen-doped porous carbon superstructures derived from hierarchical assembly of polyimide nanosheets. *Adv. Mater.* **28**, 1981-1987 (2016).
18. Tang, C. et al. Topological defects in metal-free nanocarbon for oxygen electrocatalysis. *Adv. Mater.* **28**, 6845-6851 (2016).
19. Liu, Y. et al. Elemental superdoping of graphene and carbon nanotubes. *Nat. Commun.* **7**, 10921 (2016).
20. Lai, Q., Zhao, Y., Liang, Y., He, J. & Chen, J. In situ confinement pyrolysis transformation of ZIF-8 to nitrogen-enriched meso-microporous carbon frameworks for oxygen reduction. *Adv. Funct. Mater.* **26**, 8334-8344 (2016).
21. Cui, X. et al. Pyridinic-nitrogen-dominated graphene aerogels with Fe–N–C coordination for

highly efficient oxygen reduction reaction. *Adv. Funct. Mater.* **26**, 5708-5717 (2016).
22. Graglia, M., Pampel, J., Hantke, T., Fellinger, T.-P. & Esposito, D. Nitro lignin-derived nitrogen-doped carbon as an efficient and sustainable electrocatalyst for oxygen reduction. *ACS Nano* **10**, 4364-4371 (2016).
23. Liang, H.-W., Zhuang, X., Brüller, S., Feng, X. & Müllen, K. Hierarchically porous carbons with optimized nitrogen doping as highly active electrocatalysts for oxygen reduction. *Nat. Commun.* **5**, 4973 (2014).
24. Tang, J. et al. Synthesis of nitrogen-doped mesoporous carbon spheres with extra-large pores through assembly of diblock copolymer micelles. *Angew. Chem. Int. Ed.* **54**, 588-593 (2015).
25. He, Y. et al. Highly efficient electrocatalysts for oxygen reduction reaction based on 1D ternary doped porous carbons derived from carbon nanotube directed conjugated microporous polymers. *Adv. Funct. Mater.* **26**, 8255-8265 (2016).
26. Zhang, C. et al. A nitrogen-doped ordered mesoporous carbon/graphene framework as bifunctional electrocatalyst for oxygen reduction and evolution reactions. *Nano Energy* **30**, 503-510 (2016).
27. Ye, L., Chai, G. & Wen, Z. Zn-MOF-74 derived N-doped mesoporous carbon as pH-universal electrocatalyst for oxygen reduction reaction. *Adv. Funct. Mater.* **27**, 1606190 (2017).
28. Hu, C. & Dai, L. Multifunctional carbon-based metal-free electrocatalysts for simultaneous oxygen reduction, oxygen evolution, and hydrogen evolution. *Adv. Mater.* **29**, 1604942 (2017).
29. Chai, G.-L. et al. Active sites engineering leads to exceptional ORR and OER bifunctionality in P,N Co-doped graphene frameworks. *Energy Environ. Sci.* **10**, 1186-1195 (2017).
30. Zhang, J. et al. N,P-codoped carbon networks as efficient metal-free bifunctional catalysts for oxygen reduction and hydrogen evolution reactions. *Angew. Chem. Int. Ed.* **55**, 2230-2234 (2016).
31. Yang, J. et al. A Highly efficient metal-free oxygen reduction electrocatalyst assembled from carbon nanotubes and graphene. *Adv. Mater.* **28**, 4606-4613 (2016).
32. Su, Y. et al. Sulfur-enriched conjugated polymer nanosheet derived sulfur and nitrogen co-doped porous carbon nanosheets as electrocatalysts for oxygen reduction reaction and zinc–air battery. *Adv. Funct. Mater.* **26**, 5893-5902 (2016).
33. Zhang, J., Zhao, Z., Xia, Z. & Dai, L. A metal-free bifunctional electrocatalyst for oxygen reduction and oxygen evolution reactions. *Nat. Nano.* **10**, 444-452 (2015).
34. Zhang, W., Wu, Z.-Y., Jiang, H.-L. & Yu, S.-H. Nanowire-directed templating synthesis of metal–organic framework nanofibers and their derived porous doped carbon nanofibers for enhanced electrocatalysis. *J. Am. Chem. Soc.* **136**, 14385-14388 (2014).
35. Yang, W., Fellinger, T.-P. & Antonietti, M. Efficient metal-free oxygen reduction in alkaline medium on high-surface-area mesoporous nitrogen-doped carbons made from ionic liquids and nucleobases. *J. Am. Chem. Soc.* **133**, 206-209 (2011).
36. Ai, K., Liu, Y., Ruan, C., Lu, L. & Lu, G. Sp^2 C-dominant N-doped carbon sub-micrometer spheres with a tunable size: a versatile platform for highly efficient oxygen-reduction catalysts. *Adv. Mater.* **25**, 998-1003 (2013).
37. Wei, W. et al. Nitrogen-doped carbon nanosheets with size-defined mesopores as highly efficient metal-free catalyst for the oxygen reduction reaction. *Angew. Chem.* **126**, 1596-1600 (2014).
38. Chen, P. et al. Nitrogen-doped nanoporous carbon nanosheets derived from plant biomass: an

efficient catalyst for oxygen reduction reaction. *Energy Environ. Sci.* **7**, 4095-4103 (2014).

39. Huang, Z. et al. Three-dimensional hierarchical porous nitrogen and sulfur-codoped graphene nanosheets for oxygen reduction in both alkaline and acidic media. *ChemCatChem* **9**, 987-996 (2017).
